# Resistance Profiles and Virulence Factors of Enteric *Escherichia coli* in Chronic Kidney Disease Patients at Laquintinie Hospital in Douala, Cameroon

**DOI:** 10.1155/ijm/5624252

**Published:** 2025-09-12

**Authors:** Ballue Serges T. Dadjo, Armelle T. Mbaveng, Jean W. Marbou Takougoum, Ornella D. Tsobeng, Michael F. Kengne, Victor Kuete

**Affiliations:** Department of Biochemistry, Faculty of Science, University of Dschang, Dschang, Cameroon

**Keywords:** antibiotics, bacteria, chronic kidney disease, gastroenteritis, resistance genes, virulence factors

## Abstract

*Escherichia coli* is commonly found in human feces and is the most prevalent resistant microorganism in patients with chronic kidney disease. Several studies demonstrated that virulence factors were a major cause of the emergence of pathogenic strains of *E. coli*. This study's objective was to determine the antibiotic resistance profile, detect virulence factors, and assess the prevalence of carriage of extended-spectrum beta-lactamase (ESBL) genes in fecal *E. coli* isolates obtained from chronic kidney disease patients. This research was carried out in Laquintinie Hospital of Douala between January 2022 and December 2023. In total, 458 patients with (*n* = 197) or without (*n* = 261) chronic kidney disease and suffering from gastroenteritis constituted the total population. *E. coli* isolates were obtained by using eosin methylene blue (EMB) agar and identified by the API 20E gallery system. The Kirby–Bauer method was used to determine the isolates' antibiotic resistance profile. The simplex polymerase chain reaction (PCR) served to detect virulence factors and resistance genes. It appeared that all antibiotics tested, except nalidixic acid, presented a significant resistance (*p* < 0.05) in chronic kidney disease patients contrasted to patients without chronic kidney disease. The antibiotic susceptibility testing revealed a high level of resistance to amoxicillin (94.5%), amoxicillin–clavulanic acid (79.5%), trimethoprim/sulfamethoxazole (69.9%), and ofloxacin (65.8%) in patients with chronic kidney disease. *E. coli* isolates showed (*p* < 0.001) a significantly high rate of multidrug resistance phenotype in chronic kidney disease patients (74.0%) as compared to patients without chronic kidney disease (35.7%). According to the virulence genes detected, the most prevalent pathotype of *E. coli* was the enteropathogenic *E. coli* (40.8%; *n* = 40), followed by enterotoxigenic *E. coli* (29.6%; *n* = 29) and shiga toxin–producing *E. coli* (29.6%; *n* = 29). The screening of resistance genes in pathotypes of *E. coli* has demonstrated that *bla*_TEM_ (76.5%; *n* = 75) and *bla*_CTX-M_ (75.5%; *n* = 74) were the more frequent ESBL resistance genes encountered. This study showed that a high rate of resistance, multidrug resistance, and a high frequency of enteropathogenic *E. coli* and ESBL resistance genes in *E. coli* were most often found in chronic kidney disease patients. This high level of enteric multidrug-resistant *E. coli* in chronic kidney disease patients exposes them to hazardous antibiotic treatment and serious public health issues.

## 1. Introduction

Chronic kidney disease (CKD), considered an important public health issue, is a noncommunicable disease characterized by the continuous alteration of renal functions [1–3]. Patients with CKD often exhibit metabolic disturbances, immunocompromised status, and impaired immunocompetence due to the high production of uremic toxin and chronic inflammation, making them susceptible to various bacterial infections such as gastrointestinal infections [4–7]. Gastroenteritis is an inflammation of the intestinal mucous membranes, characterized by loose feces and vomiting [8]. It is caused by the exponential growth of gut flora pathogens such as the bacteria of the Enterobacteriaceae family. Gastroenteritis is responsible for morbidity and mortality during CKD in developing countries [9]. *Escherichia coli*, a versatile and commensal bacterium belonging to the Enterobacteriaceae family, is a habitual source of infection during kidney failure (41%–61%) and can cause diarrheal infections in immunocompromised hosts [10]. The pathogenicity and antimicrobial resistance of *E. coli* are mainly due to many factors [11]. Pathogenic *E. coli* strains have been categorized into distinct ‘pathotypes' or ‘pathovars' that can cause diarrheal or enteric illness, according to the occurrence of specific virulence factors. The enteric pathovars are as follows: enterotoxigenic *E. coli* (ETEC), enteropathogenic *E. coli* (EPEC), diffusely adherent *E. coli* (DAEC), and enterohemorrhagic or Shiga toxin–producing *E. coli* (EHEC/STEC) [12]. Antimicrobial resistance of *E. coli* against clinically relevant antibiotics has been increasingly reported worldwide. These resistance phenotypes are mainly attributed to the production of special enzymes, such as extended-spectrum beta-lactamases (ESBLs) mediating resistance to the majority of beta-lactams like aztreonam (ATM), cephalosporins, and penicillins [11]. The number of ESBL resistance genes is growing exponentially in Gram-negative isolates [13]. The producers of ESBL mainly harbor *bla*_TEM_, *bla*_OXA_, *bla*_SHV_, and *bla*_CTX-M_ genes [14]. *E. coli* is still the predominant multidrug-resistant microorganism in patients with renal failure, possibly due to the high infection rates, exposure to frequent antibiotics, and hospitalizations [15, 16]. *E. coli* was considered multidrug-resistant (MDR) if it showed resistance to three or more families of antibiotics [16]. Compared to the healthy population, CKD patients are disproportionately altered by antibiotic resistance, making the growing issue of this resistance especially pertinent to them [17]. Consequently, this study's goal was to determine antibiotic resistance profiles, detect virulence factors, and assess the prevalence of carriage of ESBL genes in fecal *E. coli* isolates obtained from chronic kidney disease patients at the Laquintinie Hospital in Douala, Cameroon.

## 2. Materials and Methods

### 2.1. Study Area

The study subjects were 458 patients with gastroenteritis received at Laquintinie Hospital in Douala, Cameroon. Between January 2022 and December 2023, an observational study contrasting patients with and without CKD was conducted. The study included patients with and without chronic renal disease who had gastroenteritis symptoms such as nausea, vomiting, fever, diarrhea, belly cramps, and discomfort. The other inclusion criteria were patients who had a physician-requested bacteriological analysis of stool and who had not taken antimicrobial therapy in the earlier 14 days. Excluded patients involved maternal individuals, injured persons, patients on dialysis therapy, renal transplant recipients, and participants receiving immunosuppressant treatment.

### 2.2. Data Collection and Sampling Procedure

The methodology utilized in this investigation is identical to that used by Kengne et al. [18]. Stool samples were collected from patients who agreed to participate in the study and demonstrated symptoms of gastroenteritis. Duplicates were systematically removed. In this investigation, 458 stool samples were collected aseptically and processed within 2 h of arrival. The patient collected the feces as soon as they passed, using a sterile pot provided by us. This involved scraping the fecal waste with the given spatula and depositing it in a sterilized pot by the patient. Patients carefully cleansed their hands with soap and water prior to collection. The remaining feces were flushed down the toilet. The sample was promptly returned to the laboratory for microbiological investigation.

### 2.3. Isolation and Identification of *E. coli* From Fecal Samples

Fecal samples from symptomatic gastroenteritis collected in sterile containers were used to isolate *E. coli* bacteria. Each sample was diluted in sterile saline water, inoculated in an eosin methylene blue (EMB Levine Agar, Liofilchem, Via Scozia) petri dish, and cultivated for 24 h at 37°C. Based on morphological identification, the small, flat, purple colony with a dazzling shine was like *E. coli*. Following the primary culture, the colonies were purified through sub-culturing in freshly prepared Muller–Hinton agar medium, which was then incubated at 37°C for 24 h. The API 20E gallery identification system, which is based on 20 biochemical characters (API 20E, Biomérieux, Lyon, France), was used to confirm the identification of *E. coli*. The *E. coli* pathotypes (EPEC, ETEC, and STEC) were detected by utilization of simplex PCR.

### 2.4. Antimicrobial Susceptibility Testing

The antibiotic resistance of *E. coli* isolates was determined using the Kirby–Bauer method. According to the guidelines of the European Committee on Antimicrobial Susceptibility Testing (EUCAST), the interpretation was made using the following antimicrobials: amoxicillin + clavulanic acid (AMC, 10/5 *μ*g), amoxicillin (AMX, 10 *μ*g), ceftriaxone (CRO, 30 *μ*g), cefotaxime (CTX, 5 *μ*g), cefepime (FEP, 50 *μ*g), ATM (50 *μ*g), imipenem (IMP, 10 *μ*g), ciprofloxacin (CIP, 5 *μ*g), nalidixic acid (NAL, 30 *μ*g), ofloxacin (OFX, 2 *μ*g), sulfamethoxazole/trimetoprim (SXT, 23.75/1.25 *μ*g), fosfomycin (FOS, 200 *μ*g), gentamicin (GEN, 50 *μ*g), and amikacin (AMK, 30 *μ*g), all obtained from Singapore Biosciences, Singapore. To ensure quality control, antibiotic discs from Oxoid, Cheshire, United Kingdom, were tested using *E. coli* ATCC 25922 [19]. Multidrug resistance of *E. coli* isolates was considered if they showed resistance to three or more families of antibiotics [16].

### 2.5. DNA Extraction

Three colonies of an overnight culture were suspended in Eppendorf tubes containing 400 *μ*L of 1x Tris-EDTA buffer (10 mM Tris-Cl, 1 mM EDTA, pH 7.8) and vortexed for 5 s. Bacterial suspensions were heated at 95°C for 25 min and then centrifuged for 5 min at 13,000 rpm. For further molecular analyses, the supernatant fluid containing DNA was diluted tenfold and stored at −20°C [20].

### 2.6. Screening of Virulence Genes by Simplex PCR

Simplex PCR testing was employed to detect the pathotypes (EPEC, STEC, and ETEC) using specific primers (Table 1) to identify the virulence markers, including *elt* for the heat-labile enterotoxin genes of ETEC, the EPEC plasmid-encoded bundle-forming pilus (*bfpA* gene), and the verotoxin (*VTcom*) gene for STEC. The reaction mixture for the experiment consisted of 14.9 *μ*L of demineralized water (New England Biolab, United Kingdom); 2.5 *μ*L of 1X molecular biology buffer; 2 mM of MgCl_2_; 20 *μ*M forward primer (1.0 *μ*L) + 20 *μ*M reverse primer (1.0 *μ*L) for each of the *elt*, *VTcom*, and *bfpA* primers; 2 *μ*L of 10 mM dNTPs (New England Biolab, United Kingdom); 0.25 *μ*L of 5.0 U Taq DNA polymerase (New England Biolab, United Kingdom); and 5.0 *μ*L of DNA sample. The amplification conditions were as follows: initial DNA denaturation at 96°C for 4 min, denaturation at 95°C for 20 s, annealing at 59°C for 20 s, extension at 72°C for 1 min, and final extension at 72°C for 7 min [18, 20].

Following electrophoresis on a gel made of 2% agarose, the PCR products were marked for 25 min with ethidium bromide solution and observed using a transilluminator. As positive control strains, *E. coli* ATCC 35401 (ETEC) and ATCC 43895 (EPEC and STEC) were employed. Each of these genes controls virulence in the corresponding pathogens. EPEC was assigned to isolates that tested positive for the *bfpA* gene. Isolates that tested positive for *elt* genes were classed as ETEC [18, 20].

### 2.7. Screening for ESBL-Encoding Genes

The following antibiotic-resistant genes were identified: *bla*_TEM_, *bla*_CTX-M_, *bla*_OXA_, and *bla*_SHV_*β*-lactamases. The PCR solution for these genes involved 14.9 *μ*L of demineralized water; 2.5 *μ*L of 10x PCR buffer with 2 mM of MgCl_2_ (New England Biolab, United Kingdom); 1.0 *μ*L for every primer (20 *μ*M); 0.5 *μ*L of dNTPs (1.25 mM (New England Biolab, United Kingdom)); 0.1 *μ*L of 5.0 U Taq DNA (New England Biolab, United Kingdom); and 5.0 *μ*L of template DNA [22].  Table 2 displays the precise primer sequences and thermal profiles. After electrophoresis using a 1.5% agarose gel, the transilluminator was used to illustrate the amplified products.

### 2.8. Ethics Statement

This was done in a very precise way: first, authorization was obtained from the hospital director. Also, for the methodological procedures and guidelines applied in this research, one ethical endorsement was obtained from the National Ethics Committee for Research on Human Health of Yaoundé, Cameroon (No. 2021/12/107/CE/CNERSH/SP). Each potential participant approached was explained the purpose of the investigation and the advantages and disadvantages of participation. The informed consent certifies his/her authorization to participate in the study and guarantees the rigor of our confidentiality policy. Information such as the participant's age and sex, medical background, and any other information relevant to our study was collected.

### 2.9. Statistical Analysis

This study revealed information about the repartition of antimicrobial resistance and resistant genes in *E. coli* in different participant groups. The qualitative data was presented using frequency distribution tables. The resistance profile to antibiotics was shown as a percentage. To examine the rate of resistance of *E. coli* in patients with and without CKD, we exploited the chi-square and Fisher's exact tests. Successively, to assess the association between virulence genes, resistance genes, and the antibiotic resistance profile, the logistic regression analysis was performed. The result was considered significant if the *p* value was less than 0.05. The data was entered into Microsoft Excel and then transferred to Epi Info software, Version 7.2.4 (CDC, Atlanta, United States), to perform analysis.

## 3. Results

### 3.1. Distribution of *E. coli* Infections and Antibiotic Resistance Profile

The frequency of *E. coli* isolates among 458 patients was 37.3% (*n* = 171/458) (Table S1). The frequency of carriage in stool was distributed in CKD patients (42.7%; *n* = 73) and patients without CKD (57.3%; *n* = 98) (Table S1).

All antibiotics tested, except NAL, have presented a highly significant rate of resistance (*p* < 0.05) in patients with CKD compared to patients without CKD (Table S2 and Table 3).

### 3.2. Occurrence of Multidrug-Resistant (MDR) *E. coli* Isolates

**Figure 1:**
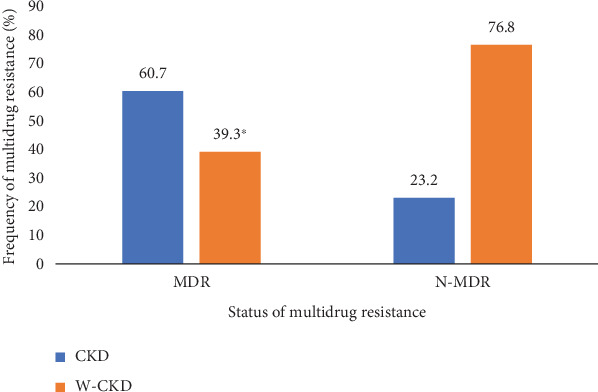
Multidrug resistance profile of *E. coli* isolates according to chronic kidney disease status. ⁣^∗^*p* < 0.001. CKD, patients with chronic kidney disease; W-CKD, patients without chronic kidney disease; MDR, multidrug resistance; N-MDR, nonmultidrug resistance.

The frequency of MDR phenotype in *E. coli* isolates was 52.1% (*n* = 89/171).  Figure 1 presents the frequency of MDR isolates from total recovered isolates (*n* = 171). *E. coli* isolates showed (*p* < 0.001) a significantly high rate of MDR in CKD patients (60.7%; *n* = 54) compared to patients without CKD (39.3%; *n* = 35) (Table S2).

### 3.3. Distribution of Pathotypes of *E. coli* Isolates

**Figure 2:**
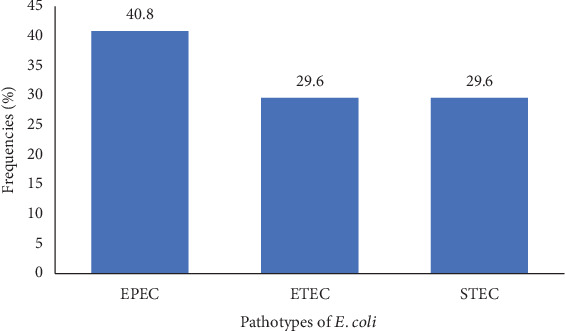
Distribution of pathotypes of *E. coli* isolates. EPEC, enteropathogenic *E. coli*; ETEC, enterotoxigenic *E. coli*; STEC, Shiga toxin–producing *E. coli.*

**Figure 3:**
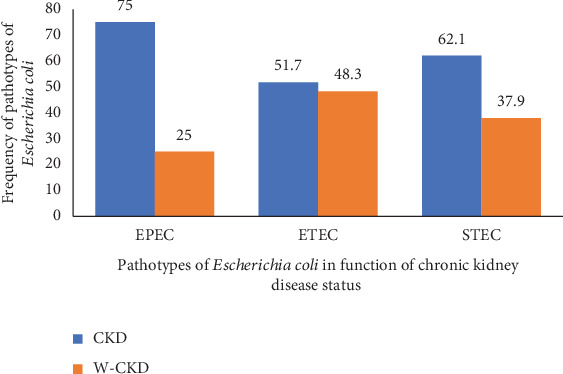
Distribution of pathotypes of *E. coli* according to chronic kidney disease status. CKD, patients with chronic kidney disease; W-CKD, patients without chronic kidney disease; EPEC, enteropathogenic *E. coli*; ETEC, enterotoxigenic *E. coli*; STEC, Shiga toxin-producing *E. coli.*

Among the 171 *E. coli* isolates obtained in this study, 98 (57.3%) isolates were identified as pathotypes of *E. coli*. According to the virulence genes detected, the most prevalent pathotype of *E. coli* detected was the EPEC (40.8%; *n* = 40/98), followed by ETEC (29.6%; *n* = 29/98) and STEC (29.6%; *n* = 29/98) (Figure 2). EPEC isolates (75% vs. 25%), ETEC (51.7% vs. 48.3%), and STEC (62.1% vs. 37.9%) were mostly obtained in CKD patients compared to patients without CKD (Table S3 and Figure 3).

### 3.4. Distribution of ESBL Resistance Genes in the Pathotypes of *E. coli*

In the 98 pathotypes of *E. coli* isolates, the prevalence of ESBL-encoding genes detected was 94.9% (*n* = 95/98). The study has demonstrated that *bla*_TEM_ (76.5%; *n* = 75) and *bla*_CTX-M_ (75.5%; *n* = 74) are the more frequent beta-lactamase genes encountered. The other beta-lactamase genes detected were *bla*_SHV_ (62.2%; *n* = 61) and *bla*_OXA_ (32.7%; *n* = 32). The *bla*_SHV_ gene was significantly more prevalent in CKD patients (73.8%; *n* = 45) as compared to patients without CKD (26.2%; *n* = 16). Also, some isolates have harbored more than one bla-resistant gene. This study has demonstrated that the more frequent combined beta-lactamase genes detected were *bla*_TEM_ + *bla*_CTX-M_ (77.3%; *n* = 58); *bla*_TEM_ + *bla*_SHV_ (66.7%; *n* = 50); and *bla*_SHV_ + *bla*_CTX-M_ (67.6%; *n* = 50) (Table S4 and Table 4).

### 3.5. Association Between Pathotypes of *E. coli* and the Beta-Lactamase Resistant Genes

The associations between pathotypes of *E. coli* and the ESBL-encoding genes are shown in Table 5. A statistically significant association (*p* < 0.05) was encountered between the occurrence of pathotypes of *E. coli* and the ESBL-encoding genes. EPEC isolates (carrying the *bfpA* gene) presented a significant association with all ESBL-encoding genes. ETEC isolates (carrying the *LT* gene) presented a significant association with *bla*_CTX-M_ (OR: 5.08; CI: 1.83–14.06; *p* < 0.001); *bla*_TEM_ (OR: 4.63; CI: 1.64–12.56; *p* = 0.001); and *bla*_SHV_ (OR: 2.51; CI: 1.10–5.71; *p* = 0.025). In addition, STEC isolates (*VTcom* carriers) showed a significant association with *bla*_TEM_ resistance genes (OR: 2.81; CI: 1.13–6.99; *p* = 0.022) and *bla*_SHV_ (OR: 2.51; CI: 1.10–5.71; *p* = 0.024) (Table S5 and Table 5).

### 3.6. Association Between Phenotypic Resistance Profile and the Pathotypes of *E. coli* Isolates

The association between the pathotypes of *E. coli* and the antimicrobial resistance profile of *E. coli* isolates is shown in Table 6. The results showed that there was a significant association between the pathotype of the EPEC and resistance to all common antibiotics (*p* < 0.001). The pathotype ETEC was significantly linked with resistance to AMX, AMC, CRO, CTX, FEP, CIP, OFX, SXT, GEN, and AMK. The pathotype STEC was significantly linked with resistance to AMX, AMC, and SXT (Table S6).

### 3.7. Association Between Carriage of the ESBL-Encoding Genes With *β*-Lactam Antibiotic Resistance in Pathotypes *E. coli* Isolates

Table 7 presents the association between ESBL-encoding genes and resistance to antibiotics belonging to the *β*-lactam family. The results show that isolates carrying the different ESBL-encoding genes (*bla*_TEM_, *bla*_SHV_, *bla*_CTX-M_, and *bla*_OXA_) show a significant association with resistance to the antibiotics AMC, AMX, ATM, CRO, CTX, and FEP. Also, there is a significant association between the carriage of *bla*_SHV_, *bla*_OXA_, and *bla*_CTX-M_ and resistance to the antibiotic IMP (Table S7).

Table 8 indicates that resistance to IMP may correlate with the concurrent presence of the *bla*_SHV_ + *bla*_CTX-M_ and *bla*_SHV_ + *bla*_OXA_ genes, alongside a positive correlation for the *bla*_TEM_ + *bla*_SHV_ genes (OR = 7.70; CI: 0.95–62.39; *p* value = 0.027). Significant associations were found between resistance to the antibiotics FEP, CTX, and CRO and the simultaneous presence of different assemblies of genes tested, except for the combination of *bla*_SHV_ + *bla*_CTX-M_. Additionally, the carriage of genes *bla*_TEM_ + *bla*_OXA_, *bla*_SHV_ + *bla*_OXA_, and *bla*_CTX-M_ + *bla*_OXA_ was associated with resistance to ATM and AMC. In addition, Table 8 shows that isolates simultaneously carrying the combinations of the *bla*_TEM_ + *bla*_CTX-M_ and *bla*_TEM_ + *bla*_SHV_ genes (OR = 13.52, CI: 1.43–127.64 and OR = 7.50, CI: 0.80–69.95, respectively) were significantly (*p* < 0.05) resistant to the antibiotic AMX (Table S7).

### 3.8. Multidrug Resistance Profile of Pathotypes of *E. coli*

**Figure 4:**
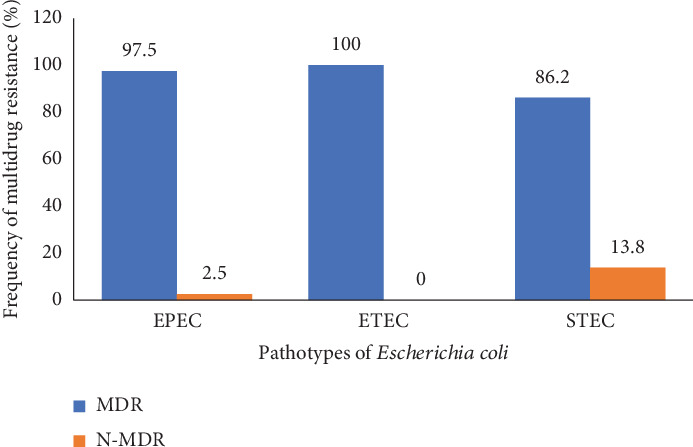
Characteristics of pathotypes of *E. coli* according to their status of multidrug resistance. MDR: multidrug resistance; EPEC: enteropathogenic *E. coli*; ETEC: enterotoxigenic *E. coli*; STEC: Shiga toxin–producing *E. coli.*

Figure 4 below shows the rate of multidrug-resistant *E. coli* pathotypes obtained from participants in this study. The pathotypes of *E. coli* according to the status of multidrug resistance show that all pathotypes of *E. coli* isolated (EPEC, ETEC, and STEC) were highly multidrug resistant to usual antibiotics (97.5%, 100%, and 86.2%, respectively) (Table S8).

## 4. Discussion

The antibiotic resistance of *E. coli* bacteria is growing in undeveloped territories and specifically in CKD patients. Thus, the multidrug resistance expressed by *E. coli* is making its infections increasingly difficult to treat. Resistance genes frequently encountered in *E. coli* bacteria, identified as the enzyme ESBL pose a significant challenge for medical interventions. Recent studies demonstrated that ESBL-producing strains of *E. coli* were highly correlated to the limitation of therapeutic options [24, 25]. Bacteria use certain molecules to colonize the host at the cellular level, virulence factors that are responsible for bacterial pathogenicity. In Douala, Cameroon, specifically in the littoral region, and among CKD patients, few authors have studied the association between pathotypes of *E. coli* and resistance genes.

This study found that 37.3% of participants (171 out of 458) carried *E. coli* in their gastrointestinal tract, with 42.7% (*n* = 73) of those having CKD and 57.3% (*n* = 98) without. *E. coli* is a prevailing Gram-negative microorganism of humans in the gastrointestinal tract and the most frequent pathogen encountered during bacterial infections [26–29]. These results corroborated those of Majeed and Aljana [30].

The study of antimicrobial resistance of *E. coli* pathogens is the best way to combat the spread of antimicrobial resistance genes. The World Health Organization presents *E. coli* as a leading pathogen due to the widespread resistance to antibiotics. In this research, a high level of resistance was noted for AMX (94.5%), AMC (79.5%), SXT (69.9%), OFX (65.8%), and FEP (59.9%). In fact, normal intestinal flora contains resistance genes, and *E. coli* isolates can efficiently exchange genetic material and plasmids with other pathogens [31, 32]. This may explain the high prevalence of drug resistance in *E. coli* isolates. In Cameroon, recent studies have revealed that antibiotics are being extensively and inappropriately used; antimicrobial agents are being dispensed over the counter without a prescription and are available to everyone [33]. The low frequency of resistance to the antibiotic IMP (21.9%) is also observed. Recent studies on the evolution of CRE (carbapenem-resistant Enterobacteriaceae) signal a potentially untreatable or very difficult-to-manage situation [34]. The resistance profile of *E. coli* isolates from stool samples of CKD and patients without CKD at the Laquintinie Hospital in Douala offers current insights into local resistance patterns and effective antimicrobial treatments for this pathogen. The *E. coli* isolates from CKD patients presented significantly higher resistance levels (*p* < 0.05) compared to patients without CKD for the antibiotics AMX (94.5% vs. 69.4%), AMC (79.5% vs. 33.7%), CRO (53.4% vs. 33.7%), CTX (56.2% vs. 38.8%), GEN (30.1% vs. 7.1%), and CIP (56.2% vs. 31.6%). The higher antibiotic resistance in CKD patients can be attributed to their increased risk of infection from multidrug-resistant organisms, frequent hospitalizations, and repeated antibiotic use, as well as their compromised immune system compared to those without CKD. Additionally, several studies have shown that *E. coli* bacteria isolated in CKD patients possess internal resistance mechanisms, including the presentation of efflux pumps and the elaboration of ESBLs [35]. These findings are consistent with those of Majeed and Aljana [30].

The screening of virulence genes in this study revealed that EPEC, ETEC, and STEC were the pathotypes of *E. coli* isolates identified. One possible explanation for these results is that EPEC, the primary pathotype to be identified, can colonize the small intestine by adhering to the mucosa. In the same line, ETEC can colonize the surface of the small bowel mucosa and elaborate enterotoxins [36–38]. These findings are consistent with those obtained in Yaoundé, Cameroon, by Fotsing-Kwetche et al. According to CKD status, EPEC was the most common pathotype of *E. coli* (40.8%), followed by ETEC (29.6%) and STEC (29.6%). EPEC isolates were regularly obtained in CKD patients (75%) as opposed to other patients. CKD is a condition that explains this result. CKD leads to the accumulation of high uremic toxin products, which affect the intestinal tract and allow exponential growth of pathogenic flora, such as the Enterobacteriaceae family. This novel environment should be favorable for mutation and development of many genetic variables, such as virulence genes identified in this research. This upper frequency of virulent genes in patients suffering from chronic disease is consistent with those obtained in Mbouda, Cameroon, and differs from those obtained in Moyo, Tanzania [39, 40].

The bacteria producers of ESBLs are within the multidrug-resistant organisms that are becoming more prevalent worldwide and posing a serious threat. To create a suitable treatment plan, it is crucial to understand the frequency and antibiotic profile of these isolates. In Cameroon, the prevalence of bacteria that produce ESBL varies. Antimicrobial agents that are currently on the market are thought to be seriously threatened by ESBL. This study documented the existence of ESBL-producing pathotypes of *E. coli*. Following molecular detection, the most common resistance genes found were *bla*_TEM_ (76.5%) and *bla*_CTX-M_ (75.5%). The prescription of third-generation cephalosporins could be the explanation for this result, as it leads to the selection of resistant mutants. Worldwide and in sub-Saharan Africa, the *bla*_CTX-M_ resistance genes are known to be disseminated in Enterobacteriales [34]. Several studies, including those by Azargun et al., Nikolié et al., Djuikoue et al., and Fils et al. in Congo, Brazzaville, as well as Kpoda et al. in Burkina Faso, have shown similar results [41–44].

Their findings showed that the repartition of ESBL genes differed significantly (*p* value < 0.05). CKD patients had a higher frequency of *bla*_SHV_ (73.8%) than those without CKD (26.2%). The overuse of antibiotics during CKD is most likely the cause of the rise in the appearance of ESBL-producing organisms [30]. The risk for infection with antibiotic-resistant *E. coli* was highly associated with antibiotic misuse [35]. These findings supported those of Majeed and Aljana [30].

The carriage of genes *bla*_TEM_, *bla*_SHV_, *bla*_CTX-M_, and *bla*_OXA_ was significantly associated with resistance to the antibiotics AMC, AMX, ATM, CRO, CTX, and FEP. *E. coli* is resistant to the majority of beta-lactam antibiotics because it can produce a wide range of beta-lactamase enzymes [37]. ESBL makers can impart resistance to beta-lactam antibiotics, including cephalosporins, aztreonam, and penicillins. Mutations that change the arrangement of amino acids surrounding the enzyme's active site give rise to these enzymes from certain genes, including *bla*_TEM_ and *bla*_SHV_ for the narrower spectrum *β*-lactamases. Plasmids that encode those genes are usually interchangeable between bacterial species [30]. These results corroborated those of Mahamat et al. [33].

Antimicrobial resistance is becoming more common in many bacterial pathogens. Antibiotic-resistant bacteria colonization and infection rates are among the highest in the world among CKD patients. Antimicrobial resistance raises the possibility of illness and mortality from infections and restricts available treatments. All isolates of pathotypes of *E. coli* detected in this study (EPEC, ETEC, and STEC) had a higher probability of being multidrug-resistant (97.5%, 100%, and 86.2%, respectively) than nonmultidrug-resistant (2.5%, 0.0%, and 13.8%, respectively). The origin and global spread of bacteria resistant to antibiotics can be attributed to several factors, including antibiotic selection pressure, transmission of resistance determinants between organisms, inadequate contagion avoidance methods, and the ease and prevalence of international travel.

The country lacks an antimicrobial stewardship program, and self-medication is prevalent. The availability of over-the-counter antibiotics, along with doctors' overreliance on antibiotics, inadequate preprescription diagnostics, and antimicrobial susceptibility testing, can also contribute to this phenomenon, leading to increased antimicrobial resistance and selective pressure on the microbiome. The high rate of multidrug-resistant *E. coli* reported in Cameroon may be attributed to the excessive and inappropriate use of antibiotics, which are easily accessible without a prescription and available over the counter [45].

The study looked at the incidence of pathotypes of ESBL-producing *E. coli* isolated from the stools of patients with chronic renal failure and discovered a significant rate of multidrug resistance to conventional antibiotics. However, to distinguish TEM, SHV, or CTX-M ESBL from other variants that are simple penicillinases, molecular research such as the sequencing of the ESBL-encoding genes identified in our work might be required [23, 46–48]. The relationship between antibiotic resistance and *E. coli* phylogenetic groups is well established. Future studies are necessary to determine the risks associated with *E. coli* enteropathogens carrying antibiotic resistance genes, their ability to transfer these genes to other bacteria in the gut microbiota, and the correlation between this antibiotic resistance and *E. coli* phylogenetic groups.

## 5. Conclusion

The results of this study showed that *E. coli* enteropathogens isolated from CKD patients exhibited high resistance to various antibiotics, including AMX (94.5%), AMC (79.5%), CTX (56.2%), FEP (59.9%), CIP (56.2%), NAL (57.5%), OFX (65.8%), and SXT (69.9%). Then, 74.0% of multidrug-resistant isolates were identified in CKD patients. The pathotype of *E. coli* identified in this research consisted of EPEC (40.8%; *n* = 40), followed by ETEC (29.6%; *n* = 29) and STEC (29.6%; *n* = 29). Concerning resistance genes, the frequency of ESBLs in *E. coli* was 94.9%. Numerous correlations have been noted between the antimicrobial resistance phenotype and resistance genes. Furthermore, there is a link between virulence factors and antibiotic resistance. All the findings highlight the significance of researching enteropathogens in relation to resistance genes and virulence factors in CKD. This antibiotic susceptibility profile allows for the adaptation of treatment approaches in CKD and provides epidemiological information regarding antibiotic resistance.

## Figures and Tables

**Table 1:** List of primers used for *E. coli* virulence typing by PCR.

**Target genes**	**Primers**	**Nucleotide sequences (5**⁣′**-3**⁣′**)**	**Amplicon size**	**PCR conditions (40 cycles)**	**Reference**
Adhesin genes					
*bfpA*/EPEC	*bfpA*–F	5⁣′-AATGGTGCTTGCGCTTGCTGC-3⁣′	326 bp	2 min at 94°C, 30 s at 92°C, 30 s at 59°C, 5 min at 72°C, 30 s at 72°C.	[21]
	*bfpA*–R	5⁣′-GCCGCTTTATCCAACCTGGTA-3⁣′			
*LT/ETEC*	*LT*–F	5⁣′-GCACACGGAGCTCCTCAGTC-3⁣′	218pb	2 min at 94°C, 30 s at 92°C, 30 s at 59°C, 5 min at 72°C, 30 s at 72°C.	
	*LT*–R	5⁣′-TCCTTCATCCTTTCAATGGCTTT-3⁣′			
Toxin genes					
*VTcom*/STEC	*VTcom*–F	5⁣′-GAGCGAAATAATTTATATGTG-3⁣′	518 bp	7 min at 94°C, 30 s at 92°C, 30 s at 60°C, 5 min at 72°C, 30 s at 72°C.	
	*VTcom*–R	5⁣′-TGATGATGGCAATTCAGTAT-3⁣′			

*Note:* All steps are repeated during the cycle.

Abbreviations: EPEC: enteropathogenic *E. coli*; ETEC: Enterotoxigenic *E. coli*; STEC: Shiga toxin–producing *E. coli.*

**Table 2:** PCR primers used for the determination of ESBL genes.

**ESBL genes**	**Primer sequence (5**⁣′**-3**⁣′**)**	**Product size (bp)**	**PCR conditions (35 cycles)**	**References**
*bla_TEM_*	F: 5⁣′CGCCGCATACACTATTCTCAGAATGA3⁣′	445	7 min at 94°C, 30 s at 94°C, 30 s at 58°C, 10 min at 68°C and 1 min at 68°C.	[18, 20, 22, 23]
	R: 5⁣′ACGCTCACCGGCTCCAGATTTAT-3⁣′			
*bla_SHV_*	F: 5⁣′-CTTTATCGGCCCTCA CTCAA-3⁣′	237	7 min at 94°C, 30 s at 94°C, 30 s at 62°C, 10 min at 68°C, and 1 min at 68°C.	
	R: 5⁣′-AGGTGCTCATCATGGGAAAG-3⁣′			
*bla_CTX-M_*	F: 5⁣′-ATGTGCAGYACCAGTAARGTKATGGC3⁣′	593	7 min at 94°C, 30 s at 94°C, 30 s at 55°C, 10 min at 68°C, and 1 min at 68°C.	
	R: 5⁣′-TGGGTRAARTARGTSACCAGAAYCAGCGG-3⁣′			
*bla_OXA_*	F: 5⁣′ACACAATACATATCAACTTCGC3⁣′	813	7 min at 94°C, 30 s at 94°C, 30 s at 58°C, 10 min at 68°C, and 1 min at 68°C.	
	R: 5⁣′-AGTGTGTTTAGA ATGGTGATC-3⁣′			

*Note:* All steps are repeated during the cycle.

**Table 3:** Susceptibility profile of *E. coli* isolates according to chronic kidney disease status.

**Patients, *E. coli* susceptibility profile and statistical analysis**					
**Antibiotic families**	**Antibiotics**		***Escherichia coli*** **n** = 171** (37.3%)**		**χ** ^2^ ** (** **p** ** value)**
			**CKD ** **n** = 73** (42.7%)**	**W-CKD ** **n** = 98** (57.3%)**	
Penicillins	AMX	S	4 (5.5%)	30 (30.6%)	16.58 (< 0.001)
		R	68 (94.5%)	68 (69.4%)	
	AMC	S	15 (20.5%)	65 (66.3%)	35.21 (< 0.001)
		R	58 (79.5%)	33 (33.7%)	

Cephalosporins	CRO	S	34 (46.6%)	65 (66.3%)	6.69 (0.009)
		R	39 (53.4%)	33 (33.7%)	
	CTX	S	31 (42.5%)	60 (61.2%)	6.84 (0.032)
		I	1 (1.4%)	0 (0.0%)	
		R	41 (56.2%)	38 (38.8%)	
	FEP	S	27 (37%)	69 (70.4%)	20.88 (< 0.001)
		I	3 (4.1%)	0 (0.0%)	
		R	43 (59.9%)	29 (29.5%)	

Monobactams	ATM	S	31 (42.5%)	78 (79.6%)	25.05 (< 0.001)
		I	5 (6.9%)	3 (3.1%)	
		R	37 (50.7%)	17 (17.4%)	

Carbapenems	IMP	S	55 (75.3%)	92 (93.9%)	12.46 (0.001)
		I	2 (2.7%)	0 (0.0%)	
		R	16 (21.9%)	6 (6.1%)	

Fluoroquinolones	CIP	S	29 (39.7%)	67 (68.4%)	16.12 (< 0.001)
		I	3 (4.1%)	0 (0.0%)	
		R	41 (56.2%)	31 (31.6%)	
	NAL	S	31 (42.5%)	51 (52%)	1.57 (0.215)
		R	42 (57.5%)	47 (48.0%)	
	OFX	S	24 (32.9%)	65 (66.3%)	19.42 (< 0.001)
		I	1 (1.4%)	0 (0.0%)	
		R	48 (65.8%)	33 (33.7%)	

Other antibiotics	SXT	S	17 (23.3%)	68 (69.4%)	36.44 (< 0.001)
		I	5 (6.9%)	1 (1%)	
		R	51 (69.9%)	29 (29.6%)	
	FOS	S	37 (50.7%)	92 (93.9%)	42.12 (< 0.001)
		R	36 (49.3%)	6 (6.1%)	

Aminoglycosides	GEN	S	51 (69.9%)	91 (92.9%)	15.70 (< 0.001)
		R	22 (30.1%)	7 (7.1%)	
	AMK	S	46 (63%)	89 (90.8%)	19.72 (< 0.001)
		I	1 (1.4%)	0 (0.0%)	
		R	26 (35.6%)	9 (9.2%)	

Abbreviations: AMC, amoxicillin + clavulanic acid; AMK, amikacin; AMX, amoxicillin; ATM, aztreonam; CIP, ciprofloxacin; CKD, chronic kidney disease patients; CRO, ceftriaxone; CTX, cefotaxime; FEP, cefepime; FOS, fosfomycin; GEN, gentamicin; I, intermediate; IMP, imipenem; NAL, nalidixic acid; OFX, ofloxacin; R, resistant; S, sensitive; SXT, sulfamethoxazole + trimethoprim; W-CKD, patients without chronic kidney disease.

**Table 4:** Distribution of ESBL resistance genes according to chronic kidney disease status.

**ESBL resistance genes**	**Frequency of ESBL genes**	**CKD**	**W-CKD**	**χ** ^2^ ** (** **p** ** value)**
Occurrence of each ESBL gene				
*bla_TEM_*	75 (76.5%)	46 (61.3%)	29 (38.7%)	1.21 (0.271)
*bla_SHV_*	61 (62.2%)	45 (73.8%)	16 (26.2%)	6.33 (0.011)
*bla_CTX-M_*	74 (75.5%)	50 (67.6%)	24 (32.4%)	1.41 (0.233)
*bla_OXA_*	32 (32.7%)	24 (75%)	8 (25%)	2.37 (0.123)
Occurrence of combinations of two ESBL genes				
*bla_TEM_ + bla_CTX-M_*	58 (77.3%)	36 (62.1%)	22 (37.9%)	0.05 (0.809)
*bla_TEM_ + bla_SHV_*	50 (66.7%)	34 (68%)	16 (32%)	2.81 (0.093)
*bla_TEM_ + bla_OXA_*	22 (29.3%)	14 (63.6%)	8 (36.4%)	0.06 (0.791)
*bla_SHV_ + bla_CTX-M_*	50 (67.6%)	38 (76%)	12 (24%)	5.00 (0.025)
*bla_SHV_ + bla_OXA_*	25 (78.1%)	19 (76%)	7 (24%)	0.06 (0.804)
*bla_CTX-M_+ bla_OXA_*	28 (87.5%)	21 (75%)	7 (25%)	0.00 (1.00)

Abbreviations: CKD, patients with chronic kidney disease; ESBL, extended-spectrum *β*-lactamase; W-CKD, patients without chronic kidney disease.

**Table 5:** Correlation between virulence genes and ESBL-encoding genes.

**Markers**	***bfpA/EPEC***		***LT/ETEC***		***VTcom/STEC***	
	**Odds ratio (LI–UI)**	**p** ** value**	**Odds ratio (LI–UI)**	**p** ** value**	**Odds ratio (LI–UI)**	**p** ** value**
*bla *_OXA_	4.95 (2.21–11.11)	**< 0.001**	1.69 (0.67–4.26)	0.254	1.35 (0.53–3.49)	0.529
*bla *_CTX-M_	5.59 (2.30–13.52)	**< 0.001**	5.08 (1.83–14.06)	**< 0.001**	1.23 (0.55–2.76)	0.615
*bla *_TEM_	2.44 (1.12–5.29)	**0.021**	4.63 (1.64–12.56)	**0.001**	2.81 (1.13–6.99)	**0.022**
*bla *_SHV_	2.79 (1.34–5.79)	**0.005**	2.51 (1.10-5.71)	**0.025**	2.51 (1.10–5.71)	**0.024**

*Note:* The *p* value is given at 95% CI and significant at ≤ 0.05. In bold: positive association between antibiotic resistance and the virulent gene.

Abbreviations: EPEC, enteropathogenic *E. coli*; ETEC, enterotoxigenic *E. coli*; LI, lower interval; STEC, Shiga toxin–producing *E. coli*; UI, upper interval.

**Table 6:** Association between virulence genes and resistance profiles of antibiotics.

**Antibiotics**	***bfpA*/EPEC**		***LT*/ETEC**		***VTcom*/STEC**	
	**OR (LI–UI)**	**p** ** value**	**OR (LI–UI)**	**p** ** value**	**OR (LI–UI)**	**p** ** value**
AMX	13.67 (1.81–103.36)	**0.001**	8.81 (1.16–67.22)	**0.012**	4.08 (0.92–18.10)	**0.046**
AMC	12.83 (4.31–38.17)	**< 0.001**	5.53 (1.99–15.30)	**< 0.001**	3.42 (1.37–8.51)	**0.005**
ATM	13.37 (5.76–31.03)	**< 0.001**	1.40 (0.61–3.23)	0.419	0.64 (0.25–1.61)	0.344
CRO	7.56 (3.30–17.32)	**< 0.001**	2.66 (1.17–6.07)	**0.016**	1.35 (0.60–3.01)	0.460
CTX	5.18 (2.33–11.53)	**< 0.001**	3.91 (1.62–9.44)	**0.001**	1.34 (0.60–2.90)	0.468
FEP	8.78 (3.72–20.71)	**< 0.001**	3.09 (1.34–7.15)	**0.006**	1.31 (0.58–2.92)	0.504
IMP	4.67 (1.87–11.68)	**< 0.001**	1.91 (0.68–5.38)	0.209	0.70 (0.19–2.54)	0.590
CIP	11.54 (4.69–28.34)	**< 0.001**	5.96 (2.38–14.94)	**< 0.001**	0.58 (0.25–1.36)	0.208
NAL	4.47 (1.97–10.13)	**< 0.001**	2.01 (0.87–4.62)	0.096	1.19 (0.54–2.66)	0.660
OFX	7.88 (3.24–19.19)	**< 0.001**	9.32 (3.07–28.21)	**< 0.001**	1.99 (0.87–4.52)	0.094
SXT	3.54 (1.65–7.59)	**< 0.001**	4.55 (1.82–11.35)	**< 0.001**	2.52 (1.09–5.81)	**0.026**
FOS	6.78 (3.09–14.86)	**< 0.001**	1.48 (0.61–3.57)	0.374	0.97 (0.38–2.47)	0.953
GEN	3.22 (1.40–7.44)	**0.004**	2.59 (1.04–6.46)	**0.036**	1.28 (0.47–3.48)	0.625
AMK	3.70 (1.67–8.16)	**< 0.001**	3.47 (1.46–8.19)	**0.003**	0.97 (0.36–2.60)	0.958

*Note:* The *p* value is given at 95% CI and significant at ≤ 0.05. In bold: positive association between antibiotic resistance and the virulence gene.

Abbreviations: AMC, amoxicillin + clavulanic acid; AMK, amikacin; AMX, amoxicillin; ATM, aztreonam; CIP, ciprofloxacin; CRO, ceftriaxone; CTX, cefotaxime; EPEC, enteropathogenic *E. coli*; ETEC, enterotoxigenic *E. coli*; FEP, cefepime; FOS, fosfomycin; GEN, gentamicin; IMP, imipenem; LI, lower interval; NAL, nalidixic acid; OFX, ofloxacin; OR, odds ratio; STEC, Shiga toxin–producing *E. coli*; SXT, sulfamethoxazole + trimethoprim; UI, upper interval.

**Table 7:** Evaluation of the correlation of carriage of the ESBL resistance genes with *β*-lactam antibiotic resistance in pathotypes of *E. coli* isolates.

**Antibiotics**	***bla***_**TEM**_		***bla***_**SHV**_		***bla***_**CTX-M**_		***bla***_**OXA**_	
	**OR (LI–UI)**	**p** ** value**	**OR (LI–UI)**	**p** ** value**	**OR (LI–UI)**	**p** ** value**	**OR (LI–UI)**	**p** ** value**
AMX	12.54 (4.55–34.54)	**< 0.001**	39.39 (5.24–296.09)	**< 0.001**	20.86 (6.06–71.80)	**< 0.001**	10.89 (1.43–82.68)	**0.004**
AMC	3.98 (2.09–7.55)	**< 0.001**	5.13 (2.63–9.98)	**< 0.001**	5.50 (2.85–10.60)	**< 0.001**	7.23 (2.64–19.78)	**< 0.001**
ATM	2.64 (1.31–5.31)	**0.005**	3.27 (1.67–6.40)	**< 0.001**	3.03 (1.50–6.09)	**0.001**	3.69 (1.69–8.03)	**< 0.001**
CRO	2.79 (1.47–5.32)	**0.001**	7.10 (3.59–14.02)	**< 0.001**	6.65 (3.29–13.40)	**< 0.001**	9.96 (3.80–25.55)	**< 0.001**
CTX	4.01 (2.08–7.72)	**< 0.001**	8.21 (4.11–16.37)	**< 0.001**	6.06 (3.08–11.92)	**< 0.001**	8.12 (3.14–20.95)	**< 0.001**
FEP	2.91 (1.53–5.55)	**< 0.001**	4.25 (2.23–8.13)	**< 0.001**	6.13 (3.08–12.23)	**< 0.001**	12.25 (4.44–33.81)	**< 0.001**
IMP	0.80 (0.33–1.95)	0.635	3.54 (1.37–9.14)	**0.006**	4.75 (1.54–14.63)	**0.003**	3.16 (1.23–8.10)	**0.012**

*Note:* The *p* value is given at 95% CI and significant at ≤ 0.05. In bold: positive association between *β*-lactam antibiotic resistance and the resistant gene.

Abbreviations: AMC, amoxicillin + clavulanic acid; AMX, amoxicillin; ATM, aztreonam; CRO, ceftriaxone; CTX, cefotaxime; EPEC, enteropathogenic *E. coli*; ETEC, enterotoxigenic *E. coli*; FEP, cefepime; IMP, imipenem; LI, lower interval; OR, odds ratio; STEC, Shiga toxin–producing *E. coli*; UI, upper interval.

**Table 8:** Correlation of the carriage of the double ESBL resistance genes with *β*-lactam antibiotic resistance in *E. coli* isolates.

**Antibiotics**	***bla***_**TEM**_ **+ *bla***_**CTX-M**_		***bla***_**TEM**_ **+ *bla***_**SHV**_		***bla***_**TEM**_ **+ *bla***_**OXA**_		***bla***_**SHV**_ **+ *bla***_**CTX-M**_		***bla***_**SHV**_ **+ *bla***_**OXA**_		***bla***_**CTX-M**_ **+ *bla***_**OXA**_	
	**OR (LI–UI)**	**p** ** value**	**OR (LI–UI)**	**p** ** value**	**OR (LI–UI)**	**p** ** value**	**OR (LI–UI)**	**p** ** value**	**OR (LI–UI)**	**p** ** value**	**OR (LI–UI)**	**p** ** value**
AMX	13.52 (1.43–127.64)	**0.004**	7.50 (0.80–69.95)	**0.041**	1.33 (0.14–12.54)	0.800	0.00 (NA)	0.642	0.00 (NA)	0.184	0.90 (0.07–10.37)	0.934
AMC	1.90 (0.74–4.88)	0.174	1.52 (0.63–3.61)	0.342	2.23 (0.74–6.65)	0.144	0.47 (0.09–2.34)	0.349	2.69 (0.79–9.18)	0.105	3.50 (1.08–11.32)	**0.029**
ATM	1.01 (0.39–2.56)	0.980	1.58 (0.67–3.74)	0.288	2.13 (0.83–5.44)	0.107	0.68 (0.20–2.26)	0.528	3.00 (1.12–8.01)	**0.025**	2.19 (0.89–5.35)	0.081
CRO	3.15 (1.20–8.25)	**0.017**	4.32 (1.78–10.48)	**< 0.001**	4.86 (1.64–14.45)	**0.002**	0.91 (0.25–3.32)	0.887	4.25 (1.27–14.26)	**0.014**	4.50 (1.52–13.29)	**0.004**
CTX	3.00 (1.17–7.66)	**0.019**	4.97 (2.05–12.09)	**< 0.001**	3.31 (1.12–9.82)	**0.025**	1.25 (0.33–4.62)	0.737	2.96 (0.87–10.06)	0.072	3.73 (1.26–11.02)	**0.013**
FEP	3.34 (1.27–8.76)	**0.011**	2.59 (1.11–6.05)	**0.025**	4.60 (1.55–16.66)	**0.003**	0.68 (0.19–2.47)	0.562	6.00 (1.79–20.04)	**0.001**	6.25 (1.95–20.01)	**< 0.001**
IMP	4.32 (0.52–35.37)	0.140	7.70 (0.95–62.39)	**0.027**	1.62 (0.44–5.96)	0.461	1.64 (0.32–8.28)	0.547	2.05 (0.66–6.30)	0.204	1.83 (0.64–5.20)	0.259

*Note:* The *p* value is given at 95% CI and significant at ≤ 0.05. In bold: positive association between *β*-lactam antibiotic resistance and the simultaneous carriage of resistant gene.

Abbreviations: AMC, amoxicillin + clavulanic acid; AMX, amoxicillin; ATM, aztreonam; CRO, ceftriaxone; CTX, cefotaxime; EPEC, enteropathogenic *E. coli*; ETEC, enterotoxigenic *E. coli*; FEP, cefepime; IMP, imipenem; LI, lower interval; OR, odds ratio; STEC, Shiga toxin–producing *E. coli*; UI, upper interval.

## Data Availability

The data that supports the findings of this study are available in the supporting information of this article.

## Supporting Information

Supporting Information Additional supporting information can be found online in the Supporting Information section. Table S1: *Escherichia coli* isolates in the total population. Table S2: Susceptibility profile and multidrug resistance of *Escherichia coli* isolates. Table S3: Distribution of pathotypes of *Escherichia coli* according to chronic kidney disease status. Table S4: Distribution of ESBL resistance genes according to chronic kidney disease status of pathotypes of *Escherichia coli* isolates. Table S5: Correlation between virulence genes and ESBL genes. Table S6: Relationship between antibiotic resistance profiles and virulence genes. Table S7: Profile of *β*-lactam antibiotic resistance in pathotypes of *E. coli* isolates carrying ESBL resistance genes. Table S8: The multidrug resistance status of pathotypes of *Escherichia coli* isolates.

## References

[1] Jazani N. H., Savoj J., Lustgarten M., Lau W. L., Vaziri N. D. (2019). Impact of Gut Dysbiosis on Neurohormonal Pathways in Chronic Kidney Disease. *Diseases*.

[2] Su G., Xu H., Riggi E. (2018). Association of Kidney Function with Infections by Multidrug-Resistant Organisms: An Electronic Medical Record Analysis. *Scientific Reports*.

[3] Hill N. R., Fatoba S. T., Oke J. L. (2016). Global Prevalence of Chronic Kidney Disease – A Systematic Review and Meta-Analysis. *PLoS One*.

[4] Mihai S., Codrici E., Popescu I. D. (2018). Inflammation-Related Mechanisms in Chronic Kidney Disease Prediction, Progression, and Outcome. *Journal of Immunology Research*.

[5] Guldris S. C., Parra E. G., Amenós A. C. (2017). Gut Microbiota in Chronic Kidney Disease. *Nefrología*.

[6] Vanegas Múnera J. M., Jiménez Quiceno J. N. (2019). Colonization and Risk of Infection by Multidrug-Resistant Bacteria in Hemodialysis Patients: A Topic of Concern. *Infectio*.

[7] Wijaya C., Eriata A. H., Rustawan I. N. T., Candra I. K. B. A., Budayanti N. N. S. (2023). Prevalence of Uropathogen Producing Extended Spectrum Beta Lactamase (ESBL) at Urinary Tract Infection in Chronic Kidney Disease Patients. *Journal of Clinical Microbiology and Infectious Diseases*.

[8] Al Asmari A. H., Alyahya N. M., Alshahrani G. A., Alshehri A. J., Alqahtani M. S., Almalki A. S. (2017). Gastroenteritis, Complications and Therapeutic Options, Review. *International Journal of Healthcare Sciences*.

[9] Thomas R., Panackal C., John M. (2013). Gastrointestinal Complications in Patients With Chronic Kidney Disease—A 5-Year Retrospective Study From a Tertiary Referral Center. *Renal Failure*.

[10] Keshi L., Weiwei X., Shoulin L. (2019). Analysis of Drug Resistance of Extended-Spectrum Beta-Lactamases-Producing *Escherichia coli* and *Klebsiella pneumoniae* in Children With Urinary Tract Infection. *Saudi Medical Journal*.

[11] Kaper J. B., Nataro J. P., Mobley H. L. T. (2004). Pathogenic *Escherichia coli*. *Nature Reviews Microbiology*.

[12] Kaur J. (2014). A Comprehensive Review on Metabolic Syndrome. *Cardiology Research and Practice*.

[13] Shilpakar A., Ansari M., Rai K. R., Rai G., Rai S. K. (2021). Prevalence of Multidrug-Resistant and Extended-Spectrum Beta-Lactamase Producing Gram-Negative Isolates From Clinical Samples in a Tertiary Care Hospital of Nepal. *Tropical Medicine and Health*.

[14] Ghazvini H., Taheri K., Edalati E., Sedighi M., Mirkalantari S. (2019). Virulence Factors and Antimicrobial Resistance in Uropathogenic *Escherichia coli* Strains Isolated From Cystitis and Pyelonephritis. *Turkish Journal of Medical Sciences*.

[15] Ilban O., Ilban A. (2023). The Relationship Between Renal Functions and Multi-Drug Resistant Organisms in Patients With Ventilator-Associated Pneumonia. *Marmara Medical Journal*.

[16] Magiorakos A.-P., Srinivasan A., Carey R. B. (2012). Multidrug-Resistant, Extensively Drug-Resistant and Pandrug-Resistant Bacteria: An International Expert Proposal for Interim Standard Definitions for Acquired Resistance. *Clinical Microbiology Infection*.

[17] Wang T. Z., Kodiyanplakkal R. P. L., Calfee D. P. (2019). Antimicrobial Resistance in Nephrology. *Nature Reviews Nephrology*.

[18] Kengne M. F., Mbaveng A. T., Karimo O. (2024). Frequency of Fecal Carriage of ESBL Resistance Genes in Multidrug-Resistant *Pseudomonas aeruginosa* Isolates From Cancer Patients at Laquintinie Hospital, Douala, Littoral Region, Cameroon. *International Journal of Microbiology*.

[19] Giske C. G., Turnidge J., Cantón R., Kahlmeter G. (2022). Eucast Steering Committee Update From the European Committee on Antimicrobial Susceptibility Testing (EUCAST). *Journal of Clinical Microbiology*.

[20] Kengne M. F., Mbaveng A. T., Marbou W. J. T. (2025). Antibiotic Resistance Profile of Enterovirulent *E.* coli Isolates Harboring Broad-Spectrum Beta-Lactamase Genes in Cancer Patients at the Laquintinie Hospital in Douala, Littoral Region, Cameroon. *International Journal of Microbiology*.

[21] Gómez-Duarte O. G., Bai J., Newell E. (2009). Detection of *Escherichia coli*, *Salmonella* spp., *Shigella* spp., *Yersinia enterocolitica*, *Vibrio cholerae*, and *Campylobacter* spp. Enteropathogens by 3-Reaction Multiplex Polymerase Chain Reaction. *Diagnostic Microbiology and Infectious Disease*.

[22] Tsobeng O. D., Mbaveng A. T., Kengne M. F., Dadjo B. S., Fonjou D. G., Kuete V. (2025). Detection of *bla*_TEM_, *bla*_OXA_, *bla*_CTX-M_, and *bla*_SHV_ Genes of Antibiotic Resistance in Diarrheagenic *E. coli* Causing Enteric Infection in Hypertensive Patients at Laquintinie Hospital, Littoral Region of Cameroon. *Journal of Infection and Public Health*.

[23] Dadjo B. S. T., Mbaveng A. T., Kengne M. F., Tsobeng O. D., Fonjou G. D. T., Kuete V. (2025). Prevalence of ESBL Resistance Genes and Fecal Carriage of Multidrug-Resistant *Pseudomonas aeruginosa* Isolates From Patients With Chronic Kidney Disease at the Laquintinie Hospital in Douala, Littoral Region, Cameroon. *Journal of Infection and Public Health*.

[24] Fotsing-Kwetche P. R., Kamgno J., Mouchet F. (2008). Enteroaggregative *Escherichia coli*: A Public Health Hazard in Yaoundé, Cameroon?. *International Journal of Biological Chemical Sciences*.

[25] Foka F. R. G., Michel N., Anicette C., Michel T., Hortense G. (2023). *Evolution du Profil de Résistance d’Escherichia coli aux Antibiotiques au Centre Hospitalier et Universitaire de Yaoundé de 2012 à 2021*. *Health Sciences and Disease*.

[26] Sharma S., Bhat G. K., Shenoy S. (2007). Virulence Factors and Drug Resistance in *Escherichia coli* Isolated From Extraintestinal Infections. *Indian Journal of Medical Microbiology*.

[27] Moszak M., Szulińska M., Bogdański P. (2020). You Are What You Eat—The Relationship Between Diet, Microbiota, and Metabolic Disorders—A Review. *Nutrients*.

[28] Allocati N., Masulli M., Alexeyev M. F., Di Ilio C. (2013). *Escherichia coli* in Europe: An Overview. *International Journal of Environmental Research and Public Health*.

[29] Daoud Z., Salem Sokhn E., Masri K. (2015). Corrigendum: *Escherichia coli* Isolated From Urinary Tract Infections of Lebanese Patients Between 2005 and 2012: Epidemiology and Profiles of Resistance. *Frontiers of Medicine*.

[30] Majeed H. T., Aljanaby A. A. J. (2019). Antibiotic Susceptibility Patterns and Prevalence of Some Extended Spectrum Beta-Lactamases Genes in Gram-Negative Bacteria Isolated From Patients Infected With Urinary Tract Infections in Al-Najaf City, Iraq. *Avicenna Journal of Medical Biotechnology*.

[31] Okeke I. N., Fayinka S. T., Lamikanra A. (2000). Antibiotic Resistance in *Escherichia coli* from Nigerian Students, 1986-1998. *Emerging Infectious Diseases*.

[32] Olorunmola F. O., Kolawole D. O., Lamikanra A. (2013). Antibiotic Resistance and Virulence Properties in *Escherichia coli* Strains From Cases of Urinary Tract Infections. *African Journal Infectious Diseases*.

[33] Mahamat S., Founou R. C., Founou L. L. (2024). Occurrence of Extended-Spectrum *β*-Lactamase (ESBL) and Carbapenemase-Producing *Escherichia coli* Isolated From Childhood Diarrhoea in Yaoundé, Cameroon. *BMC Microbiology*.

[34] Jamil B., Bokhari M. T., Saeed A. (2016). Bacteremia: Prevalence and Antimicrobial Resistance Profiling in Chronic Kidney Diseases and Renal Transplant Patients. *Journal of the Pakistan Medical Association*.

[35] Lacmago C. S. T., Fewou S. N. (2022). Antibiotic Resistance Pattern of Extended-Spectrum-Beta Lactamases-Producing *Escherichia coli* Isolated From Pregnant Women and Their New Born. *Journal of Experimental Biology*.

[36] Sumon A. H., Al-Mahmood M. R., Islam K. A. (2023). Multidrug Resistance Urinary Tract Infection in Chronic Kidney Disease Patients: An Observational Study. *Cureus*.

[37] Mare A. D., Ciurea C. N., Man A. (2021). Enteropathogenic *Escherichia coli*—A Summary of the Literature. *Gastroenterology Insights*.

[38] Marbou W. J. T., Jain P., Samajpati S. (2020). Profiling Virulence and Antimicrobial Resistance Markers of Enterovirulent *Escherichia coli* From Fecal Isolates of Adult Patients With Enteric Infections in West Cameroon. *Osong Public Health Research Perspective*.

[39] Moyo S. J., Maselle S. Y., Matee M. I., Langeland N., Mylvaganam H. (2007). Identification of Diarrheagenic *Escherichia coli* Isolated From Infants and Children in Dar es Salaam, Tanzania. *BMC Infectious Diseases*.

[40] Azargun R., Sadeghi M. R., Soroush Barhaghi M. H. (2018). The Prevalence of Plasmid-Mediated Quinolone Resistance and ESBL-Production in *Enterobacteriaceae* Isolated From Urinary Tract Infections. *Infectious Drug Resistance*.

[41] Nikolić E., Brandmajer T., Bokan V., Ulyashova M., Rubtsova M. (2018). Prevalence of *Escherichia coli* Resistant to Beta-Lactam Antibiotics Among Patients With Chronic Obstructive Pulmonary Disease and Urinary Tract Infection. *Tohoku Journal Experiences Medical*.

[42] Djuikoue C. I., Wega F., Dayomo A. L. (2022). Freshwater Linked Resistance Profile and Prevalence of *Escherichia coli* Producing ESBL Type CTX-M Strains in Cameroon Urban Cities. *Frontiers in Environmental Microbiology*.

[43] Mpelle F. L., Ngoyi E. N. O., AimÃ C., Nguimbi E., Moyen R., Kobawila S. C. (2019). First Report of the Types TEM, CTX-M, SHV and OXA-48 of Beta-Lactamases in *Escherichia coli*, From Brazzaville, Congo. *African Journal of Microbiology Research*.

[44] Kpoda D. S., Ajayi A., Somda M. (2018). Distribution of Resistance Genes Encoding ESBLs in Enterobacteriaceae Isolated From Biological Samples in Health Centers in Ouagadougou, Burkina Faso. *BMC Research Notes*.

[45] Bayaba S., Founou R. C., Tchouangueu F. T. (2025). High Prevalence of Multidrug Resistant and Extended-Spectrum *β*-Lactamase-Producing *Escherichia coli* and *Klebsiella pneumoniae* Isolated From Urinary Tract Infections in the West Region, Cameroon. *BMC Infectious Diseases*.

[46] Dadjo B. S. T., Kengne M. F., Tsobeng O. D., Mbaveng A. T., Kuete V. (2025). Prevalence and Resistance Profile of Enteric Bacteria Isolated in Chronic Kidney Disease Patients at Laquintinie Hospital of Douala: A Cross-Sectional Study. *Microbiology Journal*.

[47] Tsobeng O. D., Kengne M. F., Dadjo B. S. T., Kuete V. (2025). Multidrug Resistance of Bacteria Causing Enteric Infections in Patients With Arterial Hypertension at the Douala Laquintinie Hospital in Cameroon. *Asian Journal Biology Science*.

[48] Kengne M. F., Tsobeng O. D., Dadjo B. S. T., Kuete V., Mbaveng A. T. (2024). Multidrug Resistant Enteric Bacteria From Cancer Patients Admitted in Douala Laquintinie Hospital, Littoral Region of Cameroon. *Canadian Journal of Infectious Diseases and Medical Microbiology*.

