# Supplementary material for: Resistance Profiles and Virulence Factors of Enteric Escherichia coli in Chronic Kidney Disease Patients at Laquintinie Hospital in Douala, Cameroon
Source: Int J Microbiol. 2025 Sep 12;2025:5624252. doi: 10.1155/ijm/5624252 (PMC12449115; doi:10.1155/ijm/5624252)
Supplement: Supporting Information — Additional supporting information can be found online in the Supporting Information section. Table S1: Escherichia coli isolates in the total population. Table S2: Susceptibility profile and multidrug resistance of Escherichia coli isolates. Table S3: Distribution of pathotypes of Escherichia coli according to chronic kidney disease status. Table S4: Distribution of ESBL resistance genes according to chronic kidney disease status of pathotypes of Escherichia coli isolates. Table S5: Correlation between virulence genes and ESBL genes. Table S6: Relationship between antibiotic resistance profiles and virulence genes. Table S7: Profile of β-lactam antibiotic resistance in pathotypes of E. coli isolates carrying ESBL resistance genes. Table S8: The multidrug resistance status of pathotypes of Escherichia coli isolates. [file 5624252.f1.docx]

Supplementary file

| **S1. *Escherichia coli* isolates in the total population** | | |
| --- | --- | --- |
| **CODES** | **CKD_STATUS** | ***Escherichia coli*** |
| B1 | CKD | YES |
| B2 | CKD | YES |
| B3 | CKD | YES |
| B4 | CKD | NO |
| B5 | CKD | NO |
| B6 | CKD | NO |
| B7 | CKD | YES |
| B8 | CKD | NO |
| B9 | CKD | NO |
| B10 | CKD | NO |
| B11 | CKD | NO |
| B12 | CKD | YES |
| B13 | CKD | NO |
| B14 | CKD | NO |
| B15 | CKD | NO |
| B16 | CKD | NO |
| B17 | CKD | YES |
| B18 | CKD | YES |
| B19 | CKD | YES |
| B20 | CKD | YES |
| B21 | CKD | NO |
| B22 | CKD | YES |
| B23 | CKD | NO |
| B24 | CKD | YES |
| B25 | CKD | NO |
| B26 | CKD | YES |
| B27 | CKD | YES |
| B28 | CKD | NO |
| B29 | CKD | NO |
| B30 | CKD | YES |
| B31 | CKD | NO |
| B32 | CKD | YES |
| B33 | CKD | NO |
| B34 | CKD | YES |
| B35 | CKD | NO |
| B36 | CKD | NO |
| B37 | CKD | NO |
| B38 | CKD | NO |
| B39 | CKD | YES |
| B40 | CKD | YES |
| B41 | CKD | YES |
| B42 | CKD | NO |
| B43 | CKD | NO |
| B44 | CKD | YES |
| B45 | CKD | YES |
| B46 | CKD | YES |
| B47 | CKD | YES |
| B48 | CKD | NO |
| B49 | CKD | NO |
| B50 | CKD | YES |
| B51 | CKD | YES |
| B52 | CKD | YES |
| B53 | CKD | NO |
| B54 | CKD | NO |
| B55 | CKD | YES |
| B56 | CKD | NO |
| B57 | CKD | NO |
| B58 | CKD | NO |
| B59 | CKD | YES |
| B60 | CKD | NO |
| B61 | CKD | NO |
| B62 | CKD | NO |
| B63 | CKD | NO |
| B64 | CKD | NO |
| B65 | CKD | NO |
| B66 | CKD | YES |
| B67 | CKD | NO |
| B68 | CKD | NO |
| B69 | CKD | YES |
| B70 | CKD | NO |
| B71 | CKD | NO |
| B72 | CKD | NO |
| B73 | CKD | YES |
| B74 | CKD | NO |
| B75 | CKD | NO |
| B76 | CKD | NO |
| B77 | CKD | NO |
| B78 | CKD | YES |
| B79 | CKD | NO |
| B80 | CKD | NO |
| B81 | CKD | YES |
| B82 | CKD | NO |
| B83 | CKD | NO |
| B84 | CKD | YES |
| B85 | CKD | YES |
| B86 | CKD | NO |
| B87 | CKD | YES |
| B88 | CKD | NO |
| B89 | CKD | NO |
| B90 | CKD | NO |
| B91 | CKD | YES |
| B92 | CKD | YES |
| B93 | CKD | NO |
| B94 | CKD | NO |
| B95 | CKD | NO |
| B96 | CKD | NO |
| B97 | CKD | NO |
| B98 | CKD | NO |
| B99 | CKD | NO |
| B100 | CKD | NO |
| B101 | CKD | NO |
| B102 | CKD | YES |
| B103 | CKD | YES |
| B104 | CKD | NO |
| B105 | CKD | NO |
| B106 | CKD | NO |
| B107 | CKD | NO |
| B108 | CKD | NO |
| B109 | CKD | NO |
| B110 | CKD | NO |
| B111 | CKD | NO |
| B112 | CKD | NO |
| B113 | CKD | NO |
| B114 | CKD | NO |
| B115 | CKD | YES |
| B116 | CKD | YES |
| B117 | CKD | NO |
| B118 | CKD | NO |
| B119 | CKD | NO |
| B120 | CKD | NO |
| B121 | CKD | NO |
| B122 | CKD | NO |
| B123 | CKD | NO |
| B124 | CKD | YES |
| B125 | CKD | YES |
| B126 | CKD | YES |
| B127 | CKD | NO |
| B128 | CKD | NO |
| B129 | CKD | NO |
| B130 | CKD | YES |
| B131 | CKD | NO |
| B132 | CKD | YES |
| B133 | CKD | YES |
| B134 | CKD | NO |
| B135 | CKD | NO |
| B136 | CKD | YES |
| B137 | CKD | NO |
| B138 | CKD | NO |
| B139 | CKD | NO |
| B140 | CKD | YES |
| B141 | CKD | NO |
| B142 | CKD | NO |
| B143 | CKD | NO |
| B144 | CKD | YES |
| B145 | CKD | NO |
| B146 | CKD | NO |
| B147 | CKD | NO |
| B148 | CKD | YES |
| B149 | CKD | NO |
| B150 | CKD | NO |
| B151 | CKD | NO |
| B152 | CKD | NO |
| B153 | CKD | NO |
| B154 | CKD | NO |
| B155 | W-CKD | YES |
| B156 | CKD | NO |
| B157 | CKD | YES |
| B158 | CKD | NO |
| B159 | CKD | NO |
| B160 | CKD | YES |
| B161 | CKD | YES |
| B162 | CKD | NO |
| B163 | CKD | YES |
| B164 | CKD | NO |
| B165 | CKD | NO |
| B166 | CKD | NO |
| B167 | CKD | NO |
| B168 | CKD | YES |
| B169 | CKD | YES |
| B170 | CKD | NO |
| B171 | CKD | YES |
| B172 | CKD | YES |
| B173 | CKD | YES |
| B174 | CKD | YES |
| B175 | CKD | NO |
| B176 | CKD | NO |
| B177 | CKD | YES |
| B178 | CKD | NO |
| B179 | CKD | NO |
| B180 | CKD | YES |
| B181 | CKD | YES |
| B182 | CKD | NO |
| B183 | CKD | YES |
| B184 | CKD | YES |
| B185 | CKD | NO |
| B186 | CKD | NO |
| B187 | CKD | NO |
| B188 | CKD | NO |
| B189 | CKD | YES |
| B190 | CKD | YES |
| B191 | CKD | NO |
| B192 | CKD | YES |
| B193 | CKD | YES |
| B194 | CKD | YES |
| B195 | CKD | NO |
| B196 | CKD | NO |
| B197 | CKD | NO |
| D1 | W-CKD | YES |
| D2 | CKD | YES |
| D3 | W-CKD | YES |
| D4 | W-CKD | NO |
| D5 | W-CKD | NO |
| D6 | W-CKD | NO |
| D7 | W-CKD | NO |
| D8 | W-CKD | NO |
| D9 | W-CKD | YES |
| D10 | W-CKD | YES |
| D11 | W-CKD | YES |
| D12 | W-CKD | NO |
| D13 | W-CKD | NO |
| D14 | W-CKD | NO |
| D15 | W-CKD | NO |
| D16 | W-CKD | YES |
| D17 | W-CKD | NO |
| D18 | W-CKD | NO |
| D19 | W-CKD | NO |
| D20 | W-CKD | YES |
| D21 | W-CKD | YES |
| D22 | W-CKD | NO |
| D23 | W-CKD | NO |
| D24 | W-CKD | YES |
| D25 | W-CKD | YES |
| D26 | W-CKD | NO |
| D27 | W-CKD | NO |
| D28 | W-CKD | NO |
| D29 | W-CKD | NO |
| D30 | W-CKD | NO |
| D31 | W-CKD | NO |
| D32 | W-CKD | NO |
| D33 | W-CKD | NO |
| D34 | W-CKD | NO |
| D35 | W-CKD | NO |
| D36 | W-CKD | NO |
| D37 | W-CKD | NO |
| D38 | W-CKD | NO |
| D39 | W-CKD | NO |
| D40 | W-CKD | NO |
| D41 | W-CKD | NO |
| D42 | W-CKD | NO |
| D43 | W-CKD | NO |
| D44 | W-CKD | NO |
| D45 | W-CKD | NO |
| D46 | W-CKD | NO |
| D47 | W-CKD | NO |
| D48 | W-CKD | NO |
| D49 | W-CKD | NO |
| D50 | W-CKD | NO |
| D51 | W-CKD | NO |
| D52 | W-CKD | NO |
| D53 | W-CKD | NO |
| D54 | W-CKD | NO |
| D55 | W-CKD | NO |
| D56 | W-CKD | NO |
| D57 | W-CKD | NO |
| D58 | W-CKD | NO |
| D59 | W-CKD | NO |
| D60 | W-CKD | NO |
| D61 | W-CKD | NO |
| D62 | W-CKD | YES |
| D63 | W-CKD | YES |
| D64 | W-CKD | YES |
| D65 | W-CKD | YES |
| D66 | W-CKD | YES |
| D67 | W-CKD | YES |
| D68 | W-CKD | YES |
| D69 | W-CKD | YES |
| D70 | W-CKD | YES |
| D71 | W-CKD | YES |
| D72 | W-CKD | YES |
| D73 | W-CKD | YES |
| D74 | W-CKD | YES |
| D75 | W-CKD | NO |
| D76 | W-CKD | YES |
| D77 | W-CKD | YES |
| D78 | W-CKD | YES |
| D79 | W-CKD | NO |
| D80 | W-CKD | YES |
| D81 | W-CKD | YES |
| D82 | W-CKD | NO |
| D83 | W-CKD | NO |
| D84 | W-CKD | YES |
| D85 | W-CKD | YES |
| D86 | W-CKD | YES |
| D87 | W-CKD | YES |
| D88 | W-CKD | YES |
| D89 | W-CKD | YES |
| D90 | W-CKD | YES |
| D91 | W-CKD | YES |
| D92 | W-CKD | NO |
| D93 | W-CKD | NO |
| D94 | W-CKD | YES |
| D95 | W-CKD | YES |
| D96 | W-CKD | YES |
| D97 | W-CKD | YES |
| D98 | W-CKD | NO |
| D99 | W-CKD | YES |
| D100 | W-CKD | YES |
| D101 | W-CKD | YES |
| D102 | W-CKD | NO |
| D103 | W-CKD | YES |
| D104 | W-CKD | NO |
| D105 | W-CKD | NO |
| D106 | W-CKD | NO |
| D107 | W-CKD | NO |
| D108 | W-CKD | YES |
| D109 | W-CKD | NO |
| D110 | W-CKD | YES |
| D111 | W-CKD | YES |
| D112 | W-CKD | NO |
| D113 | W-CKD | YES |
| D114 | W-CKD | NO |
| D115 | W-CKD | NO |
| D116 | W-CKD | NO |
| D117 | W-CKD | YES |
| D118 | W-CKD | NO |
| D119 | W-CKD | NO |
| D120 | W-CKD | YES |
| D121 | W-CKD | YES |
| D122 | W-CKD | YES |
| D123 | W-CKD | NO |
| D124 | W-CKD | NO |
| D125 | W-CKD | YES |
| D126 | W-CKD | NO |
| D127 | W-CKD | YES |
| D128 | W-CKD | YES |
| D129 | W-CKD | NO |
| D130 | W-CKD | NO |
| D131 | W-CKD | YES |
| D132 | W-CKD | NO |
| D133 | W-CKD | YES |
| D134 | W-CKD | YES |
| D135 | W-CKD | NO |
| D136 | W-CKD | YES |
| D137 | W-CKD | NO |
| D138 | W-CKD | YES |
| D139 | W-CKD | NO |
| D140 | W-CKD | YES |
| D141 | W-CKD | YES |
| D142 | W-CKD | YES |
| D143 | W-CKD | YES |
| D144 | W-CKD | YES |
| D145 | W-CKD | NO |
| D146 | W-CKD | NO |
| D147 | W-CKD | YES |
| D148 | W-CKD | NO |
| D149 | W-CKD | NO |
| D150 | W-CKD | YES |
| D151 | W-CKD | YES |
| D152 | W-CKD | YES |
| D153 | W-CKD | YES |
| D154 | W-CKD | YES |
| D155 | W-CKD | YES |
| D156 | W-CKD | YES |
| D157 | W-CKD | YES |
| D158 | W-CKD | YES |
| D159 | W-CKD | YES |
| D160 | W-CKD | YES |
| D161 | W-CKD | NO |
| D162 | W-CKD | NO |
| D163 | W-CKD | YES |
| D164 | W-CKD | NO |
| D165 | W-CKD | NO |
| D166 | W-CKD | NO |
| D167 | W-CKD | YES |
| D168 | W-CKD | YES |
| D169 | W-CKD | NO |
| D170 | W-CKD | NO |
| D171 | W-CKD | NO |
| D172 | W-CKD | NO |
| D173 | W-CKD | YES |
| D174 | W-CKD | NO |
| D175 | W-CKD | NO |
| D176 | W-CKD | NO |
| D177 | W-CKD | NO |
| D178 | W-CKD | NO |
| D179 | W-CKD | NO |
| D180 | W-CKD | NO |
| D181 | W-CKD | YES |
| D182 | W-CKD | NO |
| D183 | W-CKD | YES |
| D184 | W-CKD | NO |
| D185 | W-CKD | NO |
| D186 | W-CKD | YES |
| D187 | W-CKD | NO |
| D188 | W-CKD | NO |
| D189 | W-CKD | NO |
| D190 | W-CKD | NO |
| D191 | W-CKD | NO |
| D192 | W-CKD | YES |
| D193 | W-CKD | NO |
| D194 | W-CKD | NO |
| D195 | W-CKD | NO |
| D196 | W-CKD | NO |
| D197 | W-CKD | NO |
| D198 | W-CKD | NO |
| D199 | W-CKD | YES |
| D200 | W-CKD | NO |
| D201 | W-CKD | NO |
| D202 | W-CKD | NO |
| D203 | W-CKD | NO |
| D204 | W-CKD | NO |
| D205 | W-CKD | NO |
| D206 | W-CKD | YES |
| D207 | W-CKD | NO |
| D208 | W-CKD | NO |
| D209 | W-CKD | NO |
| D210 | W-CKD | NO |
| D211 | W-CKD | NO |
| D212 | W-CKD | NO |
| D213 | W-CKD | NO |
| D214 | W-CKD | YES |
| D215 | W-CKD | YES |
| D216 | W-CKD | NO |
| D217 | W-CKD | NO |
| D218 | W-CKD | NO |
| D219 | W-CKD | NO |
| D220 | W-CKD | NO |
| D221 | W-CKD | NO |
| D222 | W-CKD | NO |
| D223 | W-CKD | NO |
| D224 | W-CKD | NO |
| D225 | W-CKD | NO |
| D226 | W-CKD | YES |
| D227 | W-CKD | NO |
| D228 | W-CKD | NO |
| D229 | W-CKD | NO |
| D230 | W-CKD | NO |
| D231 | W-CKD | NO |
| D232 | W-CKD | YES |
| D233 | W-CKD | YES |
| D234 | W-CKD | NO |
| D235 | W-CKD | NO |
| D236 | W-CKD | NO |
| D237 | W-CKD | NO |
| D238 | W-CKD | NO |
| D239 | W-CKD | NO |
| D240 | W-CKD | NO |
| D241 | W-CKD | YES |
| D242 | W-CKD | YES |
| D243 | W-CKD | NO |
| D244 | W-CKD | NO |
| D245 | W-CKD | NO |
| D246 | W-CKD | YES |
| D247 | W-CKD | NO |
| D248 | W-CKD | YES |
| D249 | W-CKD | YES |
| D250 | W-CKD | NO |
| D251 | W-CKD | NO |
| D252 | W-CKD | NO |
| D253 | W-CKD | NO |
| D254 | W-CKD | NO |
| D255 | W-CKD | NO |
| D256 | W-CKD | NO |
| D257 | W-CKD | NO |
| D258 | W-CKD | NO |
| D259 | W-CKD | NO |
| D260 | W-CKD | NO |
| D261 | W-CKD | NO |

**Legend**: Code with B: Chronic kidney disease patients; code with D: without chronic kidney disease patients; CKD: Chronic kidney disease; W-CKD: without chronic kidney disease.

| **S2. Susceptibility profile and multidrug-resistance of *Escherichia coli* isolates** | | | | | | | | | | | | | | | | | |
| --- | --- | --- | --- | --- | --- | --- | --- | --- | --- | --- | --- | --- | --- | --- | --- | --- | --- |
| **CODE** | **CKD**  **Status** | | **CIP** | **ATM** | **AMC** | **AMK** | **OFX** | **GEN** | **NAL** | **FEP** | **CTX** | **CRO** | **AMX** | **IMP** | **FOS** | **SXT** | **Multidrug resistance** |
| B1 | CKD | | S | I | S | I | S | S | S | S | S | S | S | S | R | R | N-MDR |
| B2 | CKD | | I | R | S | S | R | S | S | R | R | R | R | S | S | I | MDR |
| B3 | CKD | | R | S | R | S | R | R | R | I | R | R | R | I | R | R | MDR |
| B7 | CKD | | I | R | R | S | R | S | S | R | R | R | R | R | R | S | MDR |
| B12 | CKD | | R | S | S | R | R | S | R | S | S | S | R | S | S | S | MDR |
| B17 | CKD | | R | R | R | R | R | R | R | R | I | R | R | R | R | R | MDR |
| B18 | CKD | | R | R | R | R | R | R | R | R | R | R | R | S | R | R | MDR |
| B19 | CKD | | R | R | R | R | S | R | S | R | R | R | R | R | R | S | MDR |
| B20 | CKD | | R | R | R | S | R | S | R | R | R | R | R | R | S | R | MDR |
| B22 | CKD | | R | S | R | S | R | S | R | R | S | S | R | S | S | R | MDR |
| B24 | CKD | | R | R | R | S | R | S | R | R | S | S | R | S | S | R | MDR |
| B26 | CKD | | R | R | R | S | R | S | R | R | R | R | R | S | R | R | MDR |
| B27 | CKD | | S | R | R | R | S | S | S | R | R | R | R | S | R | R | MDR |
| B30 | CKD | | S | R | R | S | S | S | S | R | R | R | R | S | S | R | MDR |
| B32 | CKD | | R | R | R | R | I | R | R | R | R | R | R | R | R | S | MDR |
| B34 | CKD | | R | R | R | R | R | S | R | R | R | R | R | S | R | R | MDR |
| B39 | CKD | | R | I | R | S | R | S | R | R | R | R | R | S | R | I | MDR |
| B40 | CKD | | R | I | S | R | R | S | S | R | R | S | R | S | R | S | MDR |
| B41 | CKD | | S | R | R | R | S | S | S | S | R | S | R | S | R | I | MDR |
| B44 | CKD | | S | S | R | S | R | S | S | S | R | R | R | R | R | S | MDR |
| B45 | CKD | | R | S | R | S | R | S | R | S | R | S | R | S | R | S | MDR |
| B46 | CKD | | R | S | R | R | R | S | R | R | S | S | R | S | R | R | MDR |
| B47 | CKD | | S | S | R | R | R | R | S | S | R | S | R | S | R | R | MDR |
| B50 | CKD | | I | S | R | R | R | R | R | S | S | R | R | S | S | R | MDR |
| B51 | CKD | | R | R | S | S | R | R | R | R | R | R | R | S | R | R | MDR |
| B52 | CKD | | R | R | R | S | R | R | R | R | R | R | R | S | R | R | MDR |
| B55 | CKD | | R | R | R | S | R | S | R | R | R | R | R | S | R | S | MDR |
| B59 | CKD | | S | S | S | S | S | S | R | R | S | S | R | S | S | S | N-MDR |
| B66 | CKD | | R | R | R | S | R | S | S | S | R | R | R | S | S | R | MDR |
| B69 | CKD | | S | S | S | S | S | S | S | S | S | S | S | S | R | I | N-MDR |
| B73 | CKD | | S | R | R | S | R | S | S | R | R | R | R | R | S | S | MDR |
| B78 | CKD | | R | R | R | R | R | R | R | R | R | R | R | I | R | R | MDR |
| B81 | CKD | | S | S | R | S | S | S | S | S | S | S | R | S | S | R | N-MDR |
| B84 | CKD | | S | S | R | S | S | S | S | R | R | R | R | S | S | R | MDR |
| B85 | CKD | | S | I | S | S | S | S | S | I | S | S | R | S | S | R | N-MDR |
| B87 | CKD | | R | S | R | R | R | S | R | R | S | S | R | S | S | S | MDR |
| B91 | CKD | | S | S | R | S | S | S | S | S | S | S | R | S | R | R | MDR |
| B92 | CKD | | S | R | R | S | R | R | S | R | R | R | R | S | S | R | MDR |
| B102 | CKD | | S | R | R | S | S | S | S | R | S | S | R | S | R | R | MDR |
| B103 | CKD | | R | I | R | R | R | R | R | R | R | R | R | R | R | R | MDR |
| B115 | CKD | | R | R | R | R | R | S | S | S | S | S | R | S | R | R | MDR |
| B116 | CKD | | R | R | R | S | R | S | S | R | R | R | R | R | S | R | MDR |
| B124 | CKD | | S | R | S | S | S | S | S | S | S | S | S | S | S | S | N-MDR |
| B125 | CKD | | R | S | R | S | R | R | R | S | S | S | R | S | S | R | MDR |
| B126 | CKD | | R | R | R | S | R | R | R | R | R | R | R | S | R | R | MDR |
| B130 | CKD | | S | S | R | S | R | S | R | S | S | S | R | S | R | R | MDR |
| B132 | CKD | | R | S | R | R | R | R | R | S | R | R | R | S | S | R | MDR |
| B133 | CKD | | R | R | R | S | R | S | R | R | R | R | R | S | S | R | MDR |
| B136 | CKD | | S | S | S | S | S | S | S | S | S | S | R | S | S | R | N-MDR |
| B140 | CKD | | S | S | S | S | R | S | R | S | R | S | S | S | S | R | MDR |
| B144 | CKD | | S | R | S | S | S | R | S | S | S | S | R | S | S | S | MDR |
| B148 | CKD | | S | S | R | S | S | S | S | S | S | S | R | S | S | R | N-MDR |
| B155 | CKD | | S | R | S | S | S | S | S | R | S | S | R | S | R | R | MDR |
| B157 | CKD | | R | R | R | S | R | S | R | R | R | R | R | S | S | R | MDR |
| B160 | CKD | | S | S | R | S | S | S | S | S | S | S | R | S | S | R | MDR |
| B161 | CKD | | S | S | R | S | R | R | R | R | R | R | R | S | S | R | MDR |
| B163 | CKD | | R | R | R | R | R | R | R | R | R | R | R | R | R | R | MDR |
| B168 | CKD | | S | S | R | S | S | S | R | R | S | S | R | S | S | S | N-MDR |
| B169 | CKD | | R | R | R | S | S | S | S | R | S | S | R | S | R | R | MDR |
| B171 | CKD | | R | R | R | R | R | S | S | S | S | S | R | R | S | R | MDR |
| B172 | CKD | | R | S | R | S | R | R | R | R | R | R | R | R | S | R | MDR |
| B173 | CKD | | S | S | R | R | R | S | R | I | S | S | R | S | S | I | MDR |
| B174 | CKD | | R | R | R | R | R | S | R | R | R | R | R | S | S | R | MDR |
| B177 | CKD | | S | S | S | R | S | S | S | S | S | S | R | S | S | S | N-MDR |
| B180 | CKD | | R | S | R | S | R | R | R | R | R | R | R | S | R | R | MDR |
| B181 | CKD | | R | S | R | S | R | S | R | S | S | S | R | S | S | R | MDR |
| B183 | CKD | | S | S | S | R | S | S | R | R | S | R | R | R | S | S | MDR |
| B184 | CKD | | R | R | R | R | R | S | R | R | R | R | R | S | S | S | MDR |
| B189 | CKD | | R | S | R | R | R | R | R | S | S | S | R | R | R | R | MDR |
| B190 | CKD | | S | R | R | S | S | S | S | S | S | S | R | S | R | R | MDR |
| B192 | CKD | | R | S | R | S | S | S | R | S | R | R | R | S | S | R | MDR |
| B193 | CKD | | R | R | R | R | R | R | R | R | R | R | R | R | R | R | MDR |
| B194 | CKD | | R | R | R | S | R | S | R | R | R | R | R | R | R | R | MDR |
| D1 | W-CKD | S | | S | R | S | S | S | S | S | S | S | R | S | R | S | N-MDR |
| D2 | W-CKD | S | | S | R | R | S | S | S | R | S | S | R | S | S | S | MDR |
| D3 | W-CKD | S | | S | S | R | S | S | S | S | S | S | R | R | S | S | N-MDR |
| D9 | W-CKD | R | | S | R | R | R | R | R | R | R | R | R | S | R | R | MDR |
| D10 | W-CKD | R | | S | R | R | R | R | R | S | S | R | R | R | S | R | MDR |
| D11 | W-CKD | S | | S | S | S | S | S | S | S | S | S | S | S | S | S | N-MDR |
| D16 | W-CKD | S | | S | R | S | S | R | S | R | S | S | R | S | S | R | MDR |
| D20 | W-CKD | S | | S | R | S | S | R | S | S | S | S | S | S | S | S | N-MDR |
| D21 | W-CKD | R | | R | R | R | R | S | R | R | R | R | R | R | R | R | MDR |
| D24 | W-CKD | S | | S | R | S | R | S | S | S | S | S | R | S | S | S | N-MDR |
| D25 | W-CKD | R | | S | R | R | R | R | R | R | S | S | R | R | R | R | MDR |
| D62 | W-CKD | S | | S | S | S | S | S | S | S | R | R | R | S | S | S | N-MDR |
| D63 | W-CKD | R | | S | S | S | R | S | R | S | S | S | R | S | S | S | N-MDR |
| D64 | W-CKD | S | | S | S | S | S | S | S | S | S | S | S | S | S | S | N-MDR |
| D65 | W-CKD | R | | S | R | S | R | S | R | R | R | R | R | S | S | R | MDR |
| D66 | W-CKD | S | | S | S | S | S | S | S | S | S | S | R | S | S | S | N-MDR |
| D67 | W-CKD | S | | S | S | S | S | S | S | S | S | S | S | S | S | S | N-MDR |
| D68 | W-CKD | R | | S | S | S | R | S | R | S | R | R | R | S | S | S | N-MDR |
| D69 | W-CKD | S | | R | S | S | S | S | S | S | S | S | S | S | S | S | N-MDR |
| D70 | W-CKD | S | | S | R | S | S | S | R | R | R | R | R | S | S | S | N-MDR |
| D71 | W-CKD | S | | S | S | S | S | S | S | S | S | S | S | S | S | S | N-MDR |
| D72 | W-CKD | S | | S | S | S | S | S | S | S | S | S | R | S | S | S | N-MDR |
| D73 | W-CKD | R | | R | S | R | R | S | S | R | R | R | R | S | S | R | MDR |
| D74 | W-CKD | R | | R | R | S | R | S | R | R | R | R | R | S | S | R | MDR |
| D76 | W-CKD | S | | S | S | S | S | S | S | R | R | S | R | S | S | R | N-MDR |
| D77 | W-CKD | S | | S | S | S | S | S | R | S | S | S | R | S | S | S | N-MDR |
| D78 | W-CKD | S | | S | S | S | S | S | S | S | S | S | R | S | S | S | N-MDR |
| D80 | W-CKD | S | | S | R | S | S | R | S | S | S | S | S | S | S | S | N-MDR |
| D81 | W-CKD | R | | S | S | S | S | S | R | S | S | S | R | S | S | R | N-MDR |
| D84 | W-CKD | R | | S | R | S | R | S | R | R | R | R | R | R | R | R | MDR |
| D85 | W-CKD | S | | S | R | R | R | S | S | S | R | S | R | S | S | R | MDR |
| D86 | W-CKD | S | | S | S | S | S | S | S | S | S | S | S | S | S | S | N-MDR |
| D87 | W-CKD | S | | S | S | S | S | S | R | S | S | S | R | S | S | S | N-MDR |
| D88 | W-CKD | S | | S | S | S | S | S | S | S | S | S | S | S | S | S | N-MDR |
| D89 | W-CKD | R | | S | S | S | R | S | R | S | R | R | R | S | S | R | MDR |
| D90 | W-CKD | S | | I | R | S | S | S | R | S | S | R | R | S | S | R | MDR |
| D91 | W-CKD | R | | S | R | S | R | S | R | S | R | S | R | S | S | S | MDR |
| D94 | W-CKD | S | | S | S | S | S | S | S | S | S | S | S | S | S | S | N-MDR |
| D95 | W-CKD | R | | R | R | S | R | S | R | R | R | R | R | S | S | R | MDR |
| D96 | W-CKD | R | | R | R | S | R | S | R | R | R | R | R | S | S | S | MDR |
| D97 | W-CKD | S | | S | S | S | S | S | S | S | S | S | R | S | S | S | N-MDR |
| D99 | W-CKD | S | | S | S | S | S | S | S | S | S | S | R | S | S | S | N-MDR |
| D100 | W-CKD | S | | R | S | S | S | S | S | S | S | R | R | S | S | S | N-MDR |
| D101 | W-CKD | R | | S | R | S | R | S | R | R | R | R | R | S | S | R | MDR |
| D103 | W-CKD | S | | S | S | S | S | S | S | S | S | S | R | S | S | R | N-MDR |
| D108 | W-CKD | R | | S | S | S | R | S | R | S | S | S | R | S | S | R | N-MDR |
| D110 | W-CKD | R | | S | R | S | R | S | R | R | R | R | R | S | S | S | MDR |
| D111 | W-CKD | S | | R | S | S | S | S | S | R | R | S | S | S | S | R | MDR |
| D113 | W-CKD | S | | S | S | S | S | S | S | S | R | R | R | S | S | R | N-MDR |
| D117 | W-CKD | S | | I | S | S | S | S | S | R | R | R | R | S | S | S | N-MDR |
| D120 | W-CKD | S | | S | S | S | S | S | R | S | S | S | R | S | S | R | N-MDR |
| D121 | W-CKD | R | | S | R | R | R | R | R | R | R | R | R | S | S | R | MDR |
| D122 | W-CKD | S | | S | S | S | S | S | S | S | S | S | S | S | S | S | N-MDR |
| D125 | W-CKD | R | | R | S | S | R | S | R | R | R | R | R | S | S | S | MDR |
| D127 | W-CKD | S | | R | S | S | S | S | R | S | S | S | S | S | S | S | N-MDR |
| D128 | W-CKD | R | | S | R | S | R | S | R | R | R | R | R | S | S | R | MDR |
| D131 | W-CKD | S | | S | S | S | S | S | S | S | S | S | S | S | S | S | N-MDR |
| D133 | W-CKD | S | | S | R | S | S | S | S | S | S | R | R | S | S | R | MDR |
| D134 | W-CKD | R | | S | S | S | R | S | R | S | S | S | R | S | S | S | N-MDR |
| D136 | W-CKD | S | | S | S | S | S | S | R | S | S | S | S | S | S | S | N-MDR |
| D138 | W-CKD | S | | S | S | S | S | S | S | S | S | S | S | S | S | S | N-MDR |
| D140 | W-CKD | S | | S | S | S | S | S | S | S | S | S | S | S | S | S | N-MDR |
| D141 | W-CKD | S | | S | S | S | S | S | S | R | S | S | S | S | S | S | N-MDR |
| D142 | W-CKD | R | | S | S | S | R | S | R | S | S | S | R | S | S | R | N-MDR |
| D143 | W-CKD | S | | R | S | S | S | S | S | S | S | S | S | S | S | S | N-MDR |
| D144 | W-CKD | S | | S | S | S | S | S | S | S | S | S | R | S | S | S | N-MDR |
| D147 | W-CKD | R | | S | R | S | R | S | R | S | R | S | R | S | S | S | MDR |
| D150 | W-CKD | R | | R | R | S | R | S | R | R | R | R | R | S | S | S | MDR |
| D151 | W-CKD | R | | R | R | S | R | S | R | R | R | R | R | S | S | S | MDR |
| D152 | W-CKD | S | | S | S | S | S | S | R | S | R | R | R | S | S | S | N-MDR |
| D153 | W-CKD | S | | S | S | S | S | S | S | S | S | S | S | S | S | S | N-MDR |
| D154 | W-CKD | S | | S | S | S | S | S | S | S | S | S | S | S | S | R | N-MDR |
| D155 | W-CKD | R | | S | R | S | R | S | R | S | R | S | S | S | S | R | MDR |
| D156 | W-CKD | S | | R | S | S | S | S | R | R | R | S | S | S | S | S | N-MDR |
| D157 | W-CKD | S | | S | R | S | S | S | S | S | S | S | R | S | S | S | N-MDR |
| D158 | W-CKD | S | | S | S | S | S | S | R | S | S | S | R | S | S | S | N-MDR |
| D159 | W-CKD | S | | R | R | S | R | S | R | R | R | R | R | S | S | S | MDR |
| D160 | W-CKD | R | | R | R | S | R | S | R | R | R | R | R | S | S | R | MDR |
| D163 | W-CKD | S | | S | S | S | S | S | S | R | S | R | R | S | S | S | N-MDR |
| D167 | W-CKD | R | | S | S | S | R | S | R | R | S | S | S | S | S | S | N-MDR |
| D168 | W-CKD | R | | I | S | S | R | S | S | S | S | R | R | S | S | I | N-MDR |
| D173 | W-CKD | S | | R | R | S | S | S | S | R | R | R | R | S | S | R | MDR |
| D181 | W-CKD | R | | S | S | S | R | S | R | S | S | S | R | S | S | S | N-MDR |
| D183 | W-CKD | S | | S | S | S | S | S | S | S | S | S | S | S | S | S | N-MDR |
| D186 | W-CKD | S | | S | S | S | S | S | R | S | R | R | R | R | S | S | N-MDR |
| D192 | W-CKD | S | | S | S | S | S | S | R | S | R | S | R | S | R | S | N-MDR |
| D199 | W-CKD | S | | S | S | S | S | S | S | S | R | S | R | S | S | S | N-MDR |
| D206 | W-CKD | S | | S | S | S | S | S | R | S | S | S | S | S | S | S | N-MDR |
| D214 | W-CKD | S | | S | S | S | S | S | S | S | S | S | S | S | S | S | N-MDR |
| D215 | W-CKD | S | | S | S | S | S | S | S | S | R | S | R | S | S | S | N-MDR |
| D226 | W-CKD | S | | S | S | S | S | S | R | S | S | S | R | S | S | S | N-MDR |
| D232 | W-CKD | S | | S | S | S | S | S | S | S | R | R | R | S | S | S | N-MDR |
| D233 | W-CKD | S | | S | S | S | S | S | S | S | R | S | R | S | S | S | N-MDR |
| D241 | W-CKD | S | | S | S | S | S | S | S | S | S | S | S | S | S | S | N-MDR |
| D242 | W-CKD | S | | S | S | S | S | S | R | S | S | S | S | S | S | S | N-MDR |
| D246 | W-CKD | S | | S | S | S | S | S | S | S | S | S | S | S | S | S | N-MDR |
| D248 | W-CKD | S | | S | S | S | S | S | R | S | S | S | R | S | S | S | N-MDR |
| D249 | W-CKD | S | | S | R | S | S | S | R | S | S | S | R | S | S | S | N-MDR |

Legend: Code with B: Chronic kidney disease patients; code with D: without chronic kidney disease patients; CKD: Chronic kidney disease; W-CKD: without chronic kidney disease; S: sensitive; I : intermediate ; R: resistant; AMC: amoxicillin+ clavulanic acid ; AMX: amoxicillin; CRO: ceftriaxone ; CTX: cefotaxime ; FEP: cefepime ; ATM: aztreonam ; IMP: imipenem ; CIP: ciprofloxacin; NAL: nalidixic acid ; OFX: ofloxacin ; SXT: sulfamethazole+trimetoprim ; FOS: fosfomycin ; GEN: gentamycin; AMK: amikacin ; MDR: multidrug resistance; N-MDR: non multidrug resistance.

| **S3. Distribution of pathotypes of *Escherichia coli* according to chronic kidney disease status** | | | | | |
| --- | --- | --- | --- | --- | --- |
| **CODES** | **CKD Status** | **Virulence Factors** | **EPEC (*BfpA*)** | **STEC(*VTcom*)** | **ETEC (*LT*)** |
| B1 | CKD | NO | NO | NO | NO |
| B2 | CKD | YES | YES | NO | NO |
| B3 | CKD | YES | YES | NO | NO |
| B7 | CKD | YES | YES | NO | NO |
| B12 | CKD | YES | YES | NO | NO |
| B17 | CKD | YES | YES | NO | NO |
| B18 | CKD | YES | YES | NO | NO |
| B19 | CKD | YES | YES | NO | NO |
| B20 | CKD | YES | YES | NO | NO |
| B22 | CKD | YES | NO | YES | NO |
| B24 | CKD | YES | NO | NO | YES |
| B26 | CKD | YES | YES | NO | NO |
| B27 | CKD | YES | NO | YES | NO |
| B30 | CKD | YES | NO | YES | NO |
| B32 | CKD | YES | YES | NO | NO |
| B34 | CKD | YES | YES | NO | NO |
| B39 | CKD | YES | NO | YES | NO |
| B40 | CKD | YES | NO | NO | YES |
| B41 | CKD | YES | NO | NO | YES |
| B44 | CKD | YES | NO | YES | NO |
| B45 | CKD | YES | NO | YES | NO |
| B46 | CKD | YES | NO | NO | YES |
| B47 | CKD | YES | NO | YES | NO |
| B50 | CKD | YES | NO | NO | YES |
| B51 | CKD | YES | NO | NO | YES |
| B52 | CKD | YES | YES | NO | NO |
| B55 | CKD | YES | YES | NO | NO |
| B59 | CKD | NO | NO | NO | NO |
| B66 | CKD | YES | NO | NO | YES |
| B69 | CKD | NO | NO | NO | NO |
| B73 | CKD | YES | NO | YES | NO |
| B78 | CKD | YES | YES | NO | NO |
| B81 | CKD | NO | NO | NO | NO |
| B84 | CKD | YES | NO | YES | NO |
| B85 | CKD | NO | NO | NO | NO |
| B87 | CKD | YES | NO | YES | NO |
| B91 | CKD | YES | NO | NO | YES |
| B92 | CKD | YES | NO | NO | YES |
| B102 | CKD | YES | NO | YES | NO |
| B103 | CKD | YES | YES | NO | NO |
| B115 | CKD | YES | YES | NO | NO |
| B116 | CKD | YES | NO | NO | YES |
| B124 | CKD | NO | NO | NO | NO |
| B125 | CKD | YES | NO | YES | NO |
| B126 | CKD | YES | YES | NO | NO |
| B130 | CKD | YES | NO | YES | NO |
| B132 | CKD | YES | NO | NO | YES |
| B133 | CKD | YES | YES | NO | NO |
| B136 | CKD | NO | NO | NO | NO |
| B140 | CKD | YES | NO | NO | YES |
| B144 | CKD | YES | NO | YES | NO |
| B148 | CKD | NO | NO | NO | NO |
| B155 | CKD | YES | YES | NO | NO |
| B157 | CKD | YES | YES | NO | NO |
| B160 | CKD | YES | YES | NO | NO |
| B161 | CKD | YES | NO | YES | NO |
| B163 | CKD | YES | NO | NO | YES |
| B168 | CKD | NO | NO | NO | NO |
| B169 | CKD | YES | YES | NO | NO |
| B171 | CKD | YES | NO | NO | YES |
| B172 | CKD | YES | YES | NO | NO |
| B173 | CKD | YES | NO | YES | NO |
| B174 | CKD | YES | YES | NO | NO |
| B177 | CKD | NO | NO | NO | NO |
| B180 | CKD | YES | NO | NO | YES |
| B181 | CKD | YES | NO | YES | NO |
| B183 | CKD | YES | YES | NO | NO |
| B184 | CKD | YES | YES | NO | NO |
| B189 | CKD | YES | YES | NO | NO |
| B190 | CKD | YES | YES | NO | NO |
| B192 | CKD | YES | NO | YES | NO |
| B193 | CKD | YES | YES | NO | NO |
| B194 | CKD | YES | YES | NO | NO |
| D1 | W-CKD | NO | NO | NO | NO |
| D2 | W-CKD | YES | NO | NO | YES |
| D3 | W-CKD | NO | NO | NO | NO |
| D9 | W-CKD | YES | YES | NO | NO |
| D10 | W-CKD | YES | NO | NO | YES |
| D11 | W-CKD | NO | NO | NO | NO |
| D16 | W-CKD | YES | NO | YES | NO |
| D20 | W-CKD | NO | NO | NO | NO |
| D21 | W-CKD | YES | YES | NO | NO |
| D24 | W-CKD | YES | YES | NO | NO |
| D25 | W-CKD | YES | NO | NO | YES |
| D62 | W-CKD | NO | NO | NO | NO |
| D63 | W-CKD | NO | NO | NO | NO |
| D64 | W-CKD | NO | NO | NO | NO |
| D65 | W-CKD | YES | NO | NO | YES |
| D66 | W-CKD | NO | NO | NO | NO |
| D67 | W-CKD | NO | NO | NO | NO |
| D68 | W-CKD | YES | NO | YES | NO |
| D69 | W-CKD | NO | NO | NO | NO |
| D70 | W-CKD | YES | NO | YES | NO |
| D71 | W-CKD | NO | NO | NO | NO |
| D72 | W-CKD | NO | NO | NO | NO |
| D73 | W-CKD | YES | NO | NO | YES |
| D74 | W-CKD | YES | YES | NO | NO |
| D76 | W-CKD | NO | NO | NO | NO |
| D77 | W-CKD | NO | NO | NO | NO |
| D78 | W-CKD | NO | NO | NO | NO |
| D80 | W-CKD | NO | NO | NO | NO |
| D81 | W-CKD | NO | NO | NO | NO |
| D84 | W-CKD | YES | NO | NO | YES |
| D85 | W-CKD | YES | NO | YES | NO |
| D86 | W-CKD | NO | NO | NO | NO |
| D87 | W-CKD | NO | NO | NO | NO |
| D88 | W-CKD | NO | NO | NO | NO |
| D89 | W-CKD | YES | NO | YES | NO |
| D90 | W-CKD | YES | NO | YES | NO |
| D91 | W-CKD | YES | NO | NO | YES |
| D94 | W-CKD | NO | NO | NO | NO |
| D95 | W-CKD | YES | YES | NO | NO |
| D96 | W-CKD | YES | YES | NO | NO |
| D97 | W-CKD | NO | NO | NO | NO |
| D99 | W-CKD | NO | NO | NO | NO |
| D100 | W-CKD | NO | NO | NO | NO |
| D101 | W-CKD | YES | NO | NO | YES |
| D103 | W-CKD | NO | NO | NO | NO |
| D108 | W-CKD | YES | NO | YES | NO |
| D110 | W-CKD | YES | NO | NO | YES |
| D111 | W-CKD | YES | NO | YES | NO |
| D113 | W-CKD | NO | NO | NO | NO |
| D117 | W-CKD | NO | NO | NO | NO |
| D120 | W-CKD | YES | NO | YES | NO |
| D121 | W-CKD | YES | NO | NO | YES |
| D122 | W-CKD | NO | NO | NO | NO |
| D125 | W-CKD | YES | NO | NO | YES |
| D127 | W-CKD | NO | NO | NO | NO |
| D128 | W-CKD | YES | NO | NO | YES |
| D131 | W-CKD | NO | NO | NO | NO |
| D133 | W-CKD | YES | NO | YES | NO |
| D134 | W-CKD | NO | NO | NO | NO |
| D136 | W-CKD | NO | NO | NO | NO |
| D138 | W-CKD | NO | NO | NO | NO |
| D140 | W-CKD | NO | NO | NO | NO |
| D141 | W-CKD | NO | NO | NO | NO |
| D142 | W-CKD | NO | NO | NO | NO |
| D143 | W-CKD | NO | NO | NO | NO |
| D144 | W-CKD | NO | NO | NO | NO |
| D147 | W-CKD | YES | NO | NO | YES |
| D150 | W-CKD | YES | YES | NO | NO |
| D151 | W-CKD | YES | YES | NO | NO |
| D152 | W-CKD | NO | NO | NO | NO |
| D153 | W-CKD | NO | NO | NO | NO |
| D154 | W-CKD | NO | NO | NO | NO |
| D155 | W-CKD | YES | YES | NO | NO |
| D156 | W-CKD | NO | NO | NO | NO |
| D157 | W-CKD | NO | NO | NO | NO |
| D158 | W-CKD | NO | NO | NO | NO |
| D159 | W-CKD | YES | NO | YES | NO |
| D160 | W-CKD | YES | YES | NO | NO |
| D163 | W-CKD | NO | NO | NO | NO |
| D167 | W-CKD | NO | NO | NO | NO |
| D168 | W-CKD | NO | NO | NO | NO |
| D173 | W-CKD | YES | NO | NO | YES |
| D181 | W-CKD | NO | NO | NO | NO |
| D183 | W-CKD | NO | NO | NO | NO |
| D186 | W-CKD | NO | NO | NO | NO |
| D192 | W-CKD | NO | NO | NO | NO |
| D199 | W-CKD | NO | NO | NO | NO |
| D206 | W-CKD | NO | NO | NO | NO |
| D214 | W-CKD | NO | NO | NO | NO |
| D215 | W-CKD | NO | NO | NO | NO |
| D226 | W-CKD | NO | NO | NO | NO |
| D232 | W-CKD | NO | NO | NO | NO |
| D233 | W-CKD | NO | NO | NO | NO |
| D241 | W-CKD | NO | NO | NO | NO |
| D242 | W-CKD | NO | NO | NO | NO |
| D246 | W-CKD | NO | NO | NO | NO |
| D248 | W-CKD | NO | NO | NO | NO |
| D249 | W-CKD | NO | NO | NO | NO |

**Legend:** Code with B: patients with hronic kidney disease; code with D: patients without chronic kidney disease; CKD: Chronic kidney disease; W-CKD: without chronic kidney disease; EPEC: Enteropathogenic*E. coli;* ETEC: Enterotoxigenic *E. coli;* STEC: Shiga toxin producing *E. coli.*

| **S4. Distribution of ESBL resistance genes according to chronic kidney disease status of pathotypes of *Escherichia coli* isolates** | | | | | | |
| --- | --- | --- | --- | --- | --- | --- |
| **CODES** | **CKD_Status** | **ESBL_resistance genes** | ***bla*_TEM_** | ***bla*_OXA_** | ***bla*_CTX_M_** | ***bla*_SHV_** |
| B2 | CKD | YES | YES | YES | YES | YES |
| B3 | CKD | YES | YES | NO | YES | YES |
| B7 | CKD | YES | NO | NO | YES | NO |
| B12 | CKD | YES | YES | YES | YES | NO |
| B17 | CKD | YES | NO | NO | YES | YES |
| B18 | CKD | YES | YES | NO | YES | YES |
| B19 | CKD | YES | YES | YES | YES | YES |
| B20 | CKD | YES | YES | NO | YES | YES |
| B22 | CKD | YES | YES | YES | YES | YES |
| B24 | CKD | YES | YES | NO | YES | YES |
| B26 | CKD | YES | YES | YES | YES | YES |
| B27 | CKD | YES | YES | YES | YES | YES |
| B30 | CKD | YES | YES | YES | YES | YES |
| B32 | CKD | YES | YES | YES | YES | YES |
| B34 | CKD | YES | YES | YES | YES | YES |
| B39 | CKD | YES | YES | NO | YES | YES |
| B40 | CKD | YES | YES | NO | YES | YES |
| B41 | CKD | YES | YES | NO | YES | YES |
| B44 | CKD | YES | YES | YES | YES | YES |
| B45 | CKD | YES | YES | NO | YES | YES |
| B46 | CKD | YES | YES | NO | YES | YES |
| B47 | CKD | YES | YES | NO | YES | YES |
| B50 | CKD | YES | YES | YES | YES | YES |
| B51 | CKD | YES | YES | NO | YES | YES |
| B52 | CKD | YES | YES | NO | YES | NO |
| B55 | CKD | YES | YES | YES | YES | YES |
| B66 | CKD | YES | NO | NO | YES | YES |
| B73 | CKD | YES | YES | NO | NO | YES |
| B78 | CKD | YES | YES | YES | YES | NO |
| B84 | CKD | YES | YES | NO | NO | YES |
| B87 | CKD | YES | NO | YES | YES | NO |
| B91 | CKD | YES | YES | NO | NO | NO |
| B92 | CKD | YES | YES | NO | YES | YES |
| B102 | CKD | YES | YES | NO | NO | YES |
| B103 | CKD | YES | NO | YES | YES | YES |
| B115 | CKD | YES | YES | NO | YES | NO |
| B116 | CKD | YES | NO | YES | NO | YES |
| B125 | CKD | YES | YES | NO | YES | NO |
| B126 | CKD | YES | NO | YES | YES | YES |
| B130 | CKD | YES | YES | NO | NO | NO |
| B132 | CKD | YES | YES | NO | YES | YES |
| B133 | CKD | YES | NO | YES | NO | YES |
| B140 | CKD | YES | YES | NO | NO | NO |
| B144 | CKD | YES | YES | NO | NO | NO |
| B155 | CKD | YES | YES | NO | YES | YES |
| B157 | CKD | YES | NO | YES | YES | YES |
| B160 | CKD | YES | NO | NO | NO | YES |
| B161 | CKD | YES | NO | YES | YES | NO |
| B163 | CKD | YES | NO | YES | YES | NO |
| B169 | CKD | YES | YES | NO | YES | NO |
| B171 | CKD | YES | NO | NO | YES | NO |
| B172 | CKD | YES | NO | YES | YES | YES |
| B173 | CKD | YES | YES | NO | NO | NO |
| B174 | CKD | YES | YES | YES | NO | YES |
| B180 | CKD | YES | YES | NO | YES | YES |
| B181 | CKD | YES | NO | NO | YES | YES |
| B183 | CKD | YES | YES | NO | YES | YES |
| B184 | CKD | YES | YES | NO | YES | YES |
| B189 | CKD | YES | YES | NO | YES | YES |
| B190 | CKD | YES | YES | NO | NO | NO |
| B192 | CKD | YES | NO | NO | YES | NO |
| B193 | CKD | YES | NO | YES | YES | YES |
| B194 | CKD | YES | YES | NO | YES | YES |
| D2 | W-CKD | YES | YES | NO | YES | NO |
| D9 | W-CKD | YES | YES | YES | YES | NO |
| D10 | W-CKD | YES | YES | NO | YES | YES |
| D16 | W-CKD | YES | YES | NO | YES | YES |
| D21 | W-CKD | YES | YES | NO | YES | YES |
| D24 | W-CKD | NO | NO | NO | NO | NO |
| D25 | W-CKD | YES | YES | NO | YES | NO |
| D65 | W-CKD | YES | YES | YES | YES | YES |
| D68 | W-CKD | NO | NO | NO | NO | NO |
| D70 | W-CKD | NO | NO | NO | NO | NO |
| D73 | W-CKD | YES | YES | YES | YES | YES |
| D74 | W-CKD | YES | YES | YES | YES | YES |
| D84 | W-CKD | YES | YES | YES | YES | YES |
| D85 | W-CKD | YES | YES | NO | YES | YES |
| D89 | W-CKD | YES | YES | NO | YES | YES |
| D90 | W-CKD | YES | YES | NO | YES | YES |
| D91 | W-CKD | YES | YES | NO | YES | YES |
| D95 | W-CKD | YES | YES | NO | NO | NO |
| D96 | W-CKD | YES | NO | NO | YES | NO |
| D101 | W-CKD | YES | YES | NO | YES | NO |
| D108 | W-CKD | YES | YES | NO | NO | NO |
| D110 | W-CKD | YES | YES | YES | YES | NO |
| D111 | W-CKD | YES | YES | YES | YES | YES |
| D120 | W-CKD | YES | NO | NO | NO | NO |
| D121 | W-CKD | YES | YES | NO | YES | NO |
| D125 | W-CKD | YES | NO | NO | YES | NO |
| D128 | W-CKD | YES | YES | NO | YES | NO |
| D133 | W-CKD | YES | YES | NO | NO | YES |
| D147 | W-CKD | YES | YES | YES | NO | YES |
| D150 | W-CKD | YES | YES | NO | YES | NO |
| D151 | W-CKD | YES | YES | NO | YES | NO |
| D155 | W-CKD | YES | YES | NO | NO | NO |
| D159 | W-CKD | YES | YES | NO | NO | YES |
| D160 | W-CKD | YES | YES | NO | YES | NO |
| D173 | W-CKD | YES | YES | NO | NO | YES |

**Legend**: Code with B: Chronic kidney disease patients; code with D: non chronic kidney disease patients; CKD: Chronic kidney disease; W-CKD: without chronic kidney disease; ESBL: extended-spectrum β-lactamase

| **S5. Correlation between virulence genes and ESBL genes** | | | | | | | | | | |
| --- | --- | --- | --- | --- | --- | --- | --- | --- | --- | --- |
| **Codes** | **CKD_Status** | **ESBL_resistances**  **_genes** | ***bla*_TEM_** | ***bla*_OXA_** | ***bla*_CTX_M_** | ***bla*_SHV_** | **Virulence_Factors** | **EPEC (*BfpA*)** | **STEC (*VTcom*)** | **ETEC (*LT*)** |
| B2 | CKD | YES | YES | YES | YES | YES | YES | YES | NO | NO |
| B3 | CKD | YES | YES | NO | YES | YES | YES | YES | NO | NO |
| B7 | CKD | YES | NO | NO | YES | NO | YES | YES | NO | NO |
| B12 | CKD | YES | YES | YES | YES | NO | YES | YES | NO | NO |
| B17 | CKD | YES | NO | NO | YES | YES | YES | YES | NO | NO |
| B18 | CKD | YES | YES | NO | YES | YES | YES | YES | NO | NO |
| B19 | CKD | YES | YES | YES | YES | YES | YES | YES | NO | NO |
| B20 | CKD | YES | YES | NO | YES | YES | YES | YES | NO | NO |
| B22 | CKD | YES | YES | YES | YES | YES | YES | NO | YES | NO |
| B24 | CKD | YES | YES | NO | YES | YES | YES | NO | NO | YES |
| B26 | CKD | YES | YES | YES | YES | YES | YES | YES | NO | NO |
| B27 | CKD | YES | YES | YES | YES | YES | YES | NO | YES | NO |
| B30 | CKD | YES | YES | YES | YES | YES | YES | NO | YES | NO |
| B32 | CKD | YES | YES | YES | YES | YES | YES | YES | NO | NO |
| B34 | CKD | YES | YES | YES | YES | YES | YES | YES | NO | NO |
| B39 | CKD | YES | YES | NO | YES | YES | YES | NO | YES | NO |
| B40 | CKD | YES | YES | NO | YES | YES | YES | NO | NO | YES |
| B41 | CKD | YES | YES | NO | YES | YES | YES | NO | NO | YES |
| B44 | CKD | YES | YES | YES | YES | YES | YES | NO | YES | NO |
| B45 | CKD | YES | YES | NO | YES | YES | YES | NO | YES | NO |
| B46 | CKD | YES | YES | NO | YES | YES | YES | NO | NO | YES |
| B47 | CKD | YES | YES | NO | YES | YES | YES | NO | YES | NO |
| B50 | CKD | YES | YES | YES | YES | YES | YES | NO | NO | YES |
| B51 | CKD | YES | YES | NO | YES | YES | YES | NO | NO | YES |
| B52 | CKD | YES | YES | NO | YES | NO | YES | YES | NO | NO |
| B55 | CKD | YES | YES | YES | YES | YES | YES | YES | NO | NO |
| B66 | CKD | YES | NO | NO | YES | YES | YES | NO | NO | YES |
| B73 | CKD | YES | YES | NO | NO | YES | YES | NO | YES | NO |
| B78 | CKD | YES | YES | YES | YES | NO | YES | YES | NO | NO |
| B84 | CKD | YES | YES | NO | NO | YES | YES | NO | YES | NO |
| B87 | CKD | YES | NO | YES | YES | NO | YES | NO | YES | NO |
| B91 | CKD | YES | YES | NO | NO | NO | YES | NO | NO | YES |
| B92 | CKD | YES | YES | NO | YES | YES | YES | NO | NO | YES |
| B102 | CKD | YES | YES | NO | NO | YES | YES | NO | YES | NO |
| B103 | CKD | YES | NO | YES | YES | YES | YES | YES | NO | NO |
| B115 | CKD | YES | YES | NO | YES | NO | YES | YES | NO | NO |
| B116 | CKD | YES | NO | YES | NO | YES | YES | NO | NO | YES |
| B125 | CKD | YES | YES | NO | YES | NO | YES | NO | YES | NO |
| B126 | CKD | YES | NO | YES | YES | YES | YES | YES | NO | NO |
| B130 | CKD | YES | YES | NO | NO | NO | YES | NO | YES | NO |
| B132 | CKD | YES | YES | NO | YES | YES | YES | NO | NO | YES |
| B133 | CKD | YES | NO | YES | NO | YES | YES | YES | NO | NO |
| B140 | CKD | YES | YES | NO | NO | NO | YES | NO | NO | YES |
| B144 | CKD | YES | YES | NO | NO | NO | YES | NO | YES | NO |
| B155 | CKD | YES | YES | NO | YES | YES | YES | YES | NO | NO |
| B157 | CKD | YES | NO | YES | YES | YES | YES | YES | NO | NO |
| B160 | CKD | YES | NO | NO | NO | YES | YES | YES | NO | NO |
| B161 | CKD | YES | NO | YES | YES | NO | YES | NO | YES | NO |
| B163 | CKD | YES | NO | YES | YES | NO | YES | NO | NO | YES |
| B169 | CKD | YES | YES | NO | YES | NO | YES | YES | NO | NO |
| B171 | CKD | YES | NO | NO | YES | NO | YES | NO | NO | YES |
| B172 | CKD | YES | NO | YES | YES | YES | YES | YES | NO | NO |
| B173 | CKD | YES | YES | NO | NO | NO | YES | NO | YES | NO |
| B174 | CKD | YES | YES | YES | NO | YES | YES | YES | NO | NO |
| B180 | CKD | YES | YES | NO | YES | YES | YES | NO | NO | YES |
| B181 | CKD | YES | NO | NO | YES | YES | YES | NO | YES | NO |
| B183 | CKD | YES | YES | NO | YES | YES | YES | YES | NO | NO |
| B184 | CKD | YES | YES | NO | YES | YES | YES | YES | NO | NO |
| B189 | CKD | YES | YES | NO | YES | YES | YES | YES | NO | NO |
| B190 | CKD | YES | YES | NO | NO | NO | YES | YES | NO | NO |
| B192 | CKD | YES | NO | NO | YES | NO | YES | NO | YES | NO |
| B193 | CKD | YES | NO | YES | YES | YES | YES | YES | NO | NO |
| B194 | CKD | YES | YES | NO | YES | YES | YES | YES | NO | NO |
| D2 | W-CKD | YES | YES | NO | YES | NO | YES | NO | NO | YES |
| D9 | W-CKD | YES | YES | YES | YES | NO | YES | YES | NO | NO |
| D10 | W-CKD | YES | YES | NO | YES | YES | YES | NO | NO | YES |
| D16 | W-CKD | YES | YES | NO | YES | YES | YES | NO | YES | NO |
| D21 | W-CKD | YES | YES | NO | YES | YES | YES | YES | NO | NO |
| D24 | W-CKD | NO | NO | NO | NO | NO | YES | YES | NO | NO |
| D25 | W-CKD | YES | YES | NO | YES | NO | YES | NO | NO | YES |
| D65 | W-CKD | YES | YES | YES | YES | YES | YES | NO | NO | YES |
| D68 | W-CKD | NO | NO | NO | NO | NO | YES | NO | YES | NO |
| D70 | W-CKD | NO | NO | NO | NO | NO | YES | NO | YES | NO |
| D73 | W-CKD | YES | YES | YES | YES | YES | YES | NO | NO | YES |
| D74 | W-CKD | YES | YES | YES | YES | YES | YES | YES | NO | NO |
| D84 | W-CKD | YES | YES | YES | YES | YES | YES | NO | NO | YES |
| D85 | W-CKD | YES | YES | NO | YES | YES | YES | NO | YES | NO |
| D89 | W-CKD | YES | YES | NO | YES | YES | YES | NO | YES | NO |
| D90 | W-CKD | YES | YES | NO | YES | YES | YES | NO | YES | NO |
| D91 | W-CKD | YES | YES | NO | YES | YES | YES | NO | NO | YES |
| D95 | W-CKD | YES | YES | NO | NO | NO | YES | YES | NO | NO |
| D96 | W-CKD | YES | NO | NO | YES | NO | YES | YES | NO | NO |
| D101 | W-CKD | YES | YES | NO | YES | NO | YES | NO | NO | YES |
| D108 | W-CKD | YES | YES | NO | NO | NO | YES | NO | YES | NO |
| D110 | W-CKD | YES | YES | YES | YES | NO | YES | NO | NO | YES |
| D111 | W-CKD | YES | YES | YES | YES | YES | YES | NO | YES | NO |
| D120 | W-CKD | YES | NO | NO | NO | NO | YES | NO | YES | NO |
| D121 | W-CKD | YES | YES | NO | YES | NO | YES | NO | NO | YES |
| D125 | W-CKD | YES | NO | NO | YES | NO | YES | NO | NO | YES |
| D128 | W-CKD | YES | YES | NO | YES | NO | YES | NO | NO | YES |
| D133 | W-CKD | YES | YES | NO | NO | YES | YES | NO | YES | NO |
| D147 | W-CKD | YES | YES | YES | NO | YES | YES | NO | NO | YES |
| D150 | W-CKD | YES | YES | NO | YES | NO | YES | YES | NO | NO |
| D151 | W-CKD | YES | YES | NO | YES | NO | YES | YES | NO | NO |
| D155 | W-CKD | YES | YES | NO | NO | NO | YES | YES | NO | NO |
| D159 | W-CKD | YES | YES | NO | NO | YES | YES | NO | YES | NO |
| D160 | W-CKD | YES | YES | NO | YES | NO | YES | YES | NO | NO |
| D173 | W-CKD | YES | YES | NO | NO | YES | YES | NO | NO | YES |

**Legend**: Code with B: Chronic kidney disease patients; code with D: without chronic kidney disease patients; CKD: Chronic kidney disease; W-CKD: without chronic kidney disease; ESBL: extended-spectrum β-lactamase; EPEC: Enteropathogenic*E. coli;* ETEC: Enterotoxigenic *E. coli*; STEC: Shiga toxin producing *E. coli.*

| **S6. Relationship between antibiotic resistance profiles and virulence genes.** | | | | | | | | | | | | | | | | | | | | |
| --- | --- | --- | --- | --- | --- | --- | --- | --- | --- | --- | --- | --- | --- | --- | --- | --- | --- | --- | --- | --- |
| **CODES** | **CKD**  **Status** | **Virulence**  **Factors** | **EPEC**  **(*BfpA*)** | **STEC (*VTcom*)** | **ETEC (*LT*)** | **FEP** | | **CTX** | **CRO** | **AMX** | **AMC** | **ATM** | **IMP** | **CIP** | **AMK** | **OFX** | **GEN** | **NAL** | **FOS** | **SXT** |
| B2 | CKD | YES | YES | NO | NO | | YES | YES | YES | YES | NO | YES | NO | NO | NO | YES | NO | NO | NO | NO |
| B3 | CKD | YES | YES | NO | NO | | NO | YES | YES | YES | YES | NO | NO | YES | NO | YES | YES | YES | YES | YES |
| B7 | CKD | YES | YES | NO | NO | | YES | YES | YES | YES | YES | YES | YES | NO | NO | YES | NO | NO | YES | NO |
| B12 | CKD | YES | YES | NO | NO | | NO | NO | NO | YES | NO | NO | NO | YES | YES | YES | NO | YES | NO | NO |
| B17 | CKD | YES | YES | NO | NO | | YES | NO | YES | YES | YES | YES | YES | YES | YES | YES | YES | YES | YES | YES |
| B18 | CKD | YES | YES | NO | NO | | YES | YES | YES | YES | YES | YES | NO | YES | YES | YES | YES | YES | YES | YES |
| B19 | CKD | YES | YES | NO | NO | | YES | YES | YES | YES | YES | YES | YES | YES | YES | NO | YES | NO | YES | NO |
| B20 | CKD | YES | YES | NO | NO | | YES | YES | YES | YES | YES | YES | YES | YES | NO | YES | NO | YES | NO | YES |
| B22 | CKD | YES | NO | YES | NO | | YES | NO | NO | YES | YES | NO | NO | YES | NO | YES | NO | YES | NO | YES |
| B24 | CKD | YES | NO | NO | YES | | YES | NO | NO | YES | YES | YES | NO | YES | NO | YES | NO | YES | NO | YES |
| B26 | CKD | YES | YES | NO | NO | | YES | YES | YES | YES | YES | YES | NO | YES | NO | YES | NO | YES | YES | YES |
| B27 | CKD | YES | NO | YES | NO | | YES | YES | YES | YES | YES | YES | NO | NO | YES | NO | NO | NO | YES | YES |
| B30 | CKD | YES | NO | YES | NO | | YES | YES | YES | YES | YES | YES | NO | NO | NO | NO | NO | NO | NO | YES |
| B32 | CKD | YES | YES | NO | NO | | YES | YES | YES | YES | YES | YES | YES | YES | YES | NO | YES | YES | YES | NO |
| B34 | CKD | YES | YES | NO | NO | | YES | YES | YES | YES | YES | YES | NO | YES | YES | YES | NO | YES | YES | YES |
| B39 | CKD | YES | NO | YES | NO | | YES | YES | YES | YES | YES | NO | NO | YES | NO | YES | NO | YES | YES | NO |
| B40 | CKD | YES | NO | NO | YES | | YES | YES | NO | YES | NO | NO | NO | YES | YES | YES | NO | NO | YES | NO |
| B41 | CKD | YES | NO | NO | YES | | NO | YES | NO | YES | YES | YES | NO | NO | YES | NO | NO | NO | YES | NO |
| B44 | CKD | YES | NO | YES | NO | | NO | YES | YES | YES | YES | NO | YES | NO | NO | YES | NO | NO | YES | NO |
| B45 | CKD | YES | NO | YES | NO | | NO | YES | NO | YES | YES | NO | NO | YES | NO | YES | NO | YES | YES | NO |
| B46 | CKD | YES | NO | NO | YES | | YES | NO | NO | YES | YES | NO | NO | YES | YES | YES | NO | YES | YES | YES |
| B47 | CKD | YES | NO | YES | NO | | NO | YES | NO | YES | YES | NO | NO | NO | YES | YES | YES | NO | YES | YES |
| B50 | CKD | YES | NO | NO | YES | | NO | NO | YES | YES | YES | NO | NO | NO | YES | YES | YES | YES | NO | YES |
| B51 | CKD | YES | NO | NO | YES | | YES | YES | YES | YES | NO | YES | NO | YES | NO | YES | YES | YES | YES | YES |
| B52 | CKD | YES | YES | NO | NO | | YES | YES | YES | YES | YES | YES | NO | YES | NO | YES | YES | YES | YES | YES |
| B55 | CKD | YES | YES | NO | NO | | YES | YES | YES | YES | YES | YES | NO | YES | NO | YES | NO | YES | YES | NO |
| B66 | CKD | YES | NO | NO | YES | | NO | YES | YES | YES | YES | YES | NO | YES | NO | YES | NO | NO | NO | YES |
| B73 | CKD | YES | NO | YES | NO | | YES | YES | YES | YES | YES | YES | YES | NO | NO | YES | NO | NO | NO | NO |
| B78 | CKD | YES | YES | NO | NO | | YES | YES | YES | YES | YES | YES | NO | YES | YES | YES | YES | YES | YES | YES |
| B84 | CKD | YES | NO | YES | NO | | YES | YES | YES | YES | YES | NO | NO | NO | NO | NO | NO | NO | NO | YES |
| B87 | CKD | YES | NO | YES | NO | | YES | NO | NO | YES | YES | NO | NO | YES | YES | YES | NO | YES | NO | NO |
| B91 | CKD | YES | NO | NO | YES | | NO | NO | NO | YES | YES | NO | NO | NO | NO | NO | NO | NO | YES | YES |
| B92 | CKD | YES | NO | NO | YES | | YES | YES | YES | YES | YES | YES | NO | NO | NO | YES | YES | NO | NO | YES |
| B102 | CKD | YES | NO | YES | NO | | YES | NO | NO | YES | YES | YES | NO | NO | NO | NO | NO | NO | YES | YES |
| B103 | CKD | YES | YES | NO | NO | | YES | YES | YES | YES | YES | NO | YES | YES | YES | YES | YES | YES | YES | YES |
| B115 | CKD | YES | YES | NO | NO | | NO | NO | NO | YES | YES | YES | NO | YES | YES | YES | NO | NO | YES | YES |
| B116 | CKD | YES | NO | NO | YES | | YES | YES | YES | YES | YES | YES | YES | YES | NO | YES | NO | NO | NO | YES |
| B125 | CKD | YES | NO | YES | NO | | NO | NO | NO | YES | YES | NO | NO | YES | NO | YES | YES | YES | NO | YES |
| B126 | CKD | YES | YES | NO | NO | | YES | YES | YES | YES | YES | YES | NO | YES | NO | YES | YES | YES | YES | YES |
| B130 | CKD | YES | NO | YES | NO | | NO | NO | NO | YES | YES | NO | NO | NO | NO | YES | NO | YES | YES | YES |
| B132 | CKD | YES | NO | NO | YES | | NO | YES | YES | YES | YES | NO | NO | YES | YES | YES | YES | YES | NO | YES |
| B133 | CKD | YES | YES | NO | NO | | YES | YES | YES | YES | YES | YES | NO | YES | NO | YES | NO | YES | NO | YES |
| B140 | CKD | YES | NO | NO | YES | | NO | YES | NO | NO | NO | NO | NO | NO | NO | YES | NO | YES | NO | YES |
| B144 | CKD | YES | NO | YES | NO | | NO | NO | NO | YES | NO | YES | NO | NO | NO | NO | YES | NO | NO | NO |
| B155 | CKD | YES | YES | NO | NO | | YES | NO | NO | YES | NO | YES | NO | NO | NO | NO | NO | NO | YES | YES |
| B157 | CKD | YES | YES | NO | NO | | YES | YES | YES | YES | YES | YES | NO | YES | NO | YES | NO | YES | NO | YES |
| B160 | CKD | YES | YES | NO | NO | | NO | NO | NO | YES | YES | NO | NO | NO | NO | NO | NO | NO | NO | YES |
| B161 | CKD | YES | NO | YES | NO | | YES | YES | YES | YES | YES | NO | NO | NO | NO | YES | YES | YES | NO | YES |
| B163 | CKD | YES | NO | NO | YES | | YES | YES | YES | YES | YES | YES | YES | YES | YES | YES | YES | YES | YES | YES |
| B169 | CKD | YES | YES | NO | NO | | YES | NO | NO | YES | YES | YES | NO | YES | NO | NO | NO | NO | YES | YES |
| B171 | CKD | YES | NO | NO | YES | | NO | NO | NO | YES | YES | YES | YES | YES | YES | YES | NO | NO | NO | YES |
| B172 | CKD | YES | YES | NO | NO | | YES | YES | YES | YES | YES | NO | YES | YES | NO | YES | YES | YES | NO | YES |
| B173 | CKD | YES | NO | YES | NO | | NO | NO | NO | YES | YES | NO | NO | NO | YES | YES | NO | YES | NO | NO |
| B174 | CKD | YES | YES | NO | NO | | YES | YES | YES | YES | YES | YES | NO | YES | YES | YES | NO | YES | NO | YES |
| B180 | CKD | YES | NO | NO | YES | | YES | YES | YES | YES | YES | NO | NO | YES | NO | YES | YES | YES | YES | YES |
| B181 | CKD | YES | NO | YES | NO | | NO | NO | NO | YES | YES | NO | NO | YES | NO | YES | NO | YES | NO | YES |
| B183 | CKD | YES | YES | NO | NO | | YES | NO | YES | YES | NO | NO | YES | NO | YES | NO | NO | YES | NO | NO |
| B184 | CKD | YES | YES | NO | NO | | YES | YES | YES | YES | YES | YES | NO | YES | YES | YES | NO | YES | NO | NO |
| B189 | CKD | YES | YES | NO | NO | | NO | NO | NO | YES | YES | NO | YES | YES | YES | YES | YES | YES | YES | YES |
| B190 | CKD | YES | YES | NO | NO | | NO | NO | NO | YES | YES | YES | NO | NO | NO | NO | NO | NO | YES | YES |
| B192 | CKD | YES | NO | YES | NO | | YES | NO | YES | NO | NO | NO | YES | NO | YES | YES | YES | NO | NO | YES |
| B193 | CKD | YES | YES | NO | NO | | YES | YES | YES | YES | YES | YES | YES | YES | YES | YES | YES | YES | YES | YES |
| B194 | CKD | YES | YES | NO | NO | | YES | YES | YES | YES | YES | YES | YES | YES | NO | YES | NO | YES | YES | YES |
| D2 | W-CKD | YES | NO | NO | YES | | YES | NO | NO | YES | YES | NO | NO | NO | YES | NO | NO | NO | NO | NO |
| D9 | W-CKD | YES | YES | NO | NO | | YES | YES | YES | YES | YES | NO | NO | YES | YES | YES | YES | YES | YES | YES |
| D10 | W-CKD | YES | NO | NO | YES | | NO | NO | YES | YES | YES | NO | YES | YES | YES | YES | YES | YES | NO | YES |
| D16 | W-CKD | YES | NO | YES | NO | | YES | NO | NO | YES | YES | NO | NO | NO | NO | NO | YES | NO | NO | YES |
| D21 | W-CKD | YES | YES | NO | NO | | YES | YES | YES | YES | YES | YES | YES | YES | YES | YES | NO | YES | YES | YES |
| D24 | W-CKD | YES | YES | NO | NO | | NO | NO | NO | YES | YES | NO | NO | NO | NO | YES | NO | NO | NO | NO |
| D25 | W-CKD | YES | NO | NO | YES | | YES | NO | NO | YES | YES | NO | YES | YES | YES | YES | YES | YES | YES | YES |
| D65 | W-CKD | YES | NO | NO | YES | | YES | YES | YES | YES | YES | NO | NO | YES | NO | YES | NO | YES | NO | YES |
| D68 | W-CKD | YES | NO | YES | NO | | NO | YES | YES | YES | NO | NO | NO | YES | NO | YES | NO | YES | NO | NO |
| D70 | W-CKD | YES | NO | YES | NO | | YES | YES | YES | YES | YES | NO | NO | NO | NO | NO | NO | YES | NO | NO |
| D73 | W-CKD | YES | NO | NO | YES | | YES | YES | YES | YES | NO | YES | NO | YES | YES | YES | NO | NO | NO | YES |
| D74 | W-CKD | YES | YES | NO | NO | | YES | YES | YES | YES | YES | YES | NO | YES | NO | YES | NO | YES | NO | YES |
| D84 | W-CKD | YES | NO | NO | YES | | YES | YES | YES | YES | YES | NO | YES | YES | NO | YES | NO | YES | YES | YES |
| D85 | W-CKD | YES | NO | YES | NO | | NO | YES | NO | YES | YES | NO | NO | NO | YES | YES | NO | NO | NO | YES |
| D89 | W-CKD | YES | NO | YES | NO | | NO | YES | YES | YES | NO | NO | NO | YES | NO | YES | NO | YES | NO | YES |
| D90 | W-CKD | YES | NO | YES | NO | | NO | NO | YES | YES | YES | NO | NO | NO | NO | NO | NO | YES | NO | YES |
| D91 | W-CKD | YES | NO | NO | YES | | NO | YES | NO | YES | YES | NO | NO | YES | NO | YES | NO | YES | NO | NO |
| D95 | W-CKD | YES | YES | NO | NO | | YES | YES | YES | YES | YES | YES | NO | YES | NO | YES | NO | YES | NO | YES |
| D96 | W-CKD | YES | YES | NO | NO | | YES | YES | YES | YES | YES | YES | NO | YES | NO | YES | NO | YES | NO | NO |
| D101 | W-CKD | YES | NO | NO | YES | | YES | YES | YES | YES | YES | NO | NO | YES | NO | YES | NO | YES | NO | YES |
| D108 | W-CKD | YES | NO | YES | NO | | NO | NO | NO | YES | NO | NO | NO | YES | NO | YES | NO | YES | NO | YES |
| D110 | W-CKD | YES | NO | NO | YES | | YES | YES | YES | YES | YES | NO | NO | YES | NO | YES | NO | YES | NO | NO |
| D111 | W-CKD | YES | NO | YES | NO | | YES | YES | NO | NO | NO | YES | NO | NO | NO | NO | NO | NO | NO | YES |
| D120 | W-CKD | YES | NO | YES | NO | | NO | NO | NO | YES | NO | NO | NO | NO | NO | NO | NO | YES | NO | YES |
| D121 | W-CKD | YES | NO | NO | YES | | YES | YES | YES | YES | YES | NO | NO | YES | YES | YES | YES | YES | NO | YES |
| D125 | W-CKD | YES | NO | NO | YES | | YES | YES | YES | YES | NO | YES | NO | YES | NO | YES | NO | YES | NO | NO |
| D128 | W-CKD | YES | NO | NO | YES | | YES | YES | YES | YES | YES | NO | NO | YES | NO | YES | NO | YES | NO | YES |
| D133 | W-CKD | YES | NO | YES | NO | | NO | NO | YES | YES | YES | NO | NO | NO | NO | NO | NO | NO | NO | YES |
| D147 | W-CKD | YES | NO | NO | YES | | NO | YES | NO | YES | YES | NO | NO | YES | NO | YES | NO | YES | NO | NO |
| D150 | W-CKD | YES | YES | NO | NO | | YES | YES | YES | YES | YES | YES | NO | YES | NO | YES | NO | YES | NO | NO |
| D151 | W-CKD | YES | YES | NO | NO | | YES | YES | YES | YES | YES | YES | NO | YES | NO | YES | NO | YES | NO | NO |
| D155 | W-CKD | YES | YES | NO | NO | | NO | YES | NO | NO | YES | NO | NO | YES | NO | YES | NO | YES | NO | YES |
| D159 | W-CKD | YES | NO | YES | NO | | YES | YES | YES | YES | YES | YES | NO | NO | NO | YES | NO | YES | NO | NO |
| D160 | W-CKD | YES | YES | NO | NO | | YES | YES | YES | YES | YES | YES | NO | YES | NO | YES | NO | YES | NO | YES |
| D173 | W-CKD | YES | NO | NO | YES | | YES | YES | YES | YES | YES | YES | NO | NO | NO | NO | NO | NO | NO | YES |

**Legend**: Code with B: Chronic kidney disease patients; code with D: without chronic kidney disease patients; CKD: Chronic kidney disease; W-CKD: without chronic kidney disease; EPEC: Enteropathogenic*E. coli;* ETEC: Enterotoxigenic*E. coli;* STEC: Shiga toxin producing *E. coli,* AMC: amoxicillin+ clavulanic acid ; AMX: amoxicillin; CRO: ceftriaxon ; CTX: cefotaxim ; FEP: cefepim ; ATM: aztreonam ; IMP: imipenem ; CIP: ciprofloxacin; NAL: nalixidic acid ; OFX: ofloxacin ; SXT: sulphamethazole+trimetoprim ; FOS: fosfomycin ; GEN: gentamycin; AMK: amikacin*.*

| **S7. Profile of β-lactam antibiotic resistance in pathotypes of *E. coli* isolates carriers ESBL resistance genes.** | | | | | | | | | | | | |
| --- | --- | --- | --- | --- | --- | --- | --- | --- | --- | --- | --- | --- |
| **CODES** | **CKD_Status** | ***bla*_TEM_** | ***bla*_OXA_** | ***bla*_CTX_M_** | ***bla*_SHV_** | **FEP** | **CTX** | **CRO** | **AMX** | **AMC** | **ATM** | **IMP** |
| B2 | CKD | YES | YES | YES | YES | YES | YES | YES | YES | NO | YES | NO |
| B3 | CKD | YES | NO | YES | YES | NO | YES | YES | YES | YES | NO | NO |
| B7 | CKD | NO | NO | YES | NO | YES | YES | YES | YES | YES | YES | YES |
| B12 | CKD | YES | YES | YES | NO | NO | NO | NO | YES | NO | NO | NO |
| B17 | CKD | NO | NO | YES | YES | YES | NO | YES | YES | YES | YES | YES |
| B18 | CKD | YES | NO | YES | YES | YES | YES | YES | YES | YES | YES | NO |
| B19 | CKD | YES | YES | YES | YES | YES | YES | YES | YES | YES | YES | YES |
| B20 | CKD | YES | NO | YES | YES | YES | YES | YES | YES | YES | YES | YES |
| B22 | CKD | YES | YES | YES | YES | YES | NO | NO | YES | YES | NO | NO |
| B24 | CKD | YES | NO | YES | YES | YES | NO | NO | YES | YES | YES | NO |
| B26 | CKD | YES | YES | YES | YES | YES | YES | YES | YES | YES | YES | NO |
| B27 | CKD | YES | YES | YES | YES | YES | YES | YES | YES | YES | YES | NO |
| B30 | CKD | YES | YES | YES | YES | YES | YES | YES | YES | YES | YES | NO |
| B32 | CKD | YES | YES | YES | YES | YES | YES | YES | YES | YES | YES | YES |
| B34 | CKD | YES | YES | YES | YES | YES | YES | YES | YES | YES | YES | NO |
| B39 | CKD | YES | NO | YES | YES | YES | YES | YES | YES | YES | NO | NO |
| B40 | CKD | YES | NO | YES | YES | YES | YES | NO | YES | NO | NO | NO |
| B41 | CKD | YES | NO | YES | YES | NO | YES | NO | YES | YES | YES | NO |
| B44 | CKD | YES | YES | YES | YES | NO | YES | YES | YES | YES | NO | YES |
| B45 | CKD | YES | NO | YES | YES | NO | YES | NO | YES | YES | NO | NO |
| B46 | CKD | YES | NO | YES | YES | YES | NO | NO | YES | YES | NO | NO |
| B47 | CKD | YES | NO | YES | YES | NO | YES | NO | YES | YES | NO | NO |
| B50 | CKD | YES | YES | YES | YES | NO | NO | YES | YES | YES | NO | NO |
| B51 | CKD | YES | NO | YES | YES | YES | YES | YES | YES | NO | YES | NO |
| B52 | CKD | YES | NO | YES | NO | YES | YES | YES | YES | YES | YES | NO |
| B55 | CKD | YES | YES | YES | YES | YES | YES | YES | YES | YES | YES | NO |
| B66 | CKD | NO | NO | YES | YES | NO | YES | YES | YES | YES | YES | NO |
| B73 | CKD | YES | NO | NO | YES | YES | YES | YES | YES | YES | YES | YES |
| B78 | CKD | YES | YES | YES | NO | YES | YES | YES | YES | YES | YES | NO |
| B84 | CKD | YES | NO | NO | YES | YES | YES | YES | YES | YES | NO | NO |
| B87 | CKD | NO | YES | YES | NO | YES | NO | NO | YES | YES | NO | NO |
| B91 | CKD | YES | NO | NO | NO | NO | NO | NO | YES | YES | NO | NO |
| B92 | CKD | YES | NO | YES | YES | YES | YES | YES | YES | YES | YES | NO |
| B102 | CKD | YES | NO | NO | YES | YES | NO | NO | YES | YES | YES | NO |
| B103 | CKD | NO | YES | YES | YES | YES | YES | YES | YES | YES | NO | YES |
| B115 | CKD | YES | NO | YES | NO | NO | NO | NO | YES | YES | YES | NO |
| B116 | CKD | NO | YES | NO | YES | YES | YES | YES | YES | YES | YES | YES |
| B125 | CKD | YES | NO | YES | NO | NO | NO | NO | YES | YES | NO | NO |
| B126 | CKD | NO | YES | YES | YES | YES | YES | YES | YES | YES | YES | NO |
| B130 | CKD | YES | NO | NO | NO | NO | NO | NO | YES | YES | NO | NO |
| B132 | CKD | YES | NO | YES | YES | NO | YES | YES | YES | YES | NO | NO |
| B133 | CKD | NO | YES | NO | YES | YES | YES | YES | YES | YES | YES | NO |
| B140 | CKD | YES | NO | NO | NO | NO | YES | NO | NO | NO | NO | NO |
| B144 | CKD | YES | NO | NO | NO | NO | NO | NO | YES | NO | YES | NO |
| B155 | CKD | YES | NO | YES | YES | YES | NO | NO | YES | NO | YES | NO |
| B157 | CKD | NO | YES | YES | YES | YES | YES | YES | YES | YES | YES | NO |
| B160 | CKD | NO | NO | NO | YES | NO | NO | NO | YES | YES | NO | NO |
| B161 | CKD | NO | YES | YES | NO | YES | YES | YES | YES | YES | NO | NO |
| B163 | CKD | NO | YES | YES | NO | YES | YES | YES | YES | YES | YES | YES |
| B169 | CKD | YES | NO | YES | NO | YES | NO | NO | YES | YES | YES | NO |
| B171 | CKD | NO | NO | YES | NO | NO | NO | NO | YES | YES | YES | YES |
| B172 | CKD | NO | YES | YES | YES | YES | YES | YES | YES | YES | NO | YES |
| B173 | CKD | YES | NO | NO | NO | NO | NO | NO | YES | YES | NO | NO |
| B174 | CKD | YES | YES | NO | YES | YES | YES | YES | YES | YES | YES | NO |
| B180 | CKD | YES | NO | YES | YES | YES | YES | YES | YES | YES | NO | NO |
| B181 | CKD | NO | NO | YES | YES | NO | NO | NO | YES | YES | NO | NO |
| B183 | CKD | YES | NO | YES | YES | YES | NO | YES | YES | NO | NO | YES |
| B184 | CKD | YES | NO | YES | YES | YES | YES | YES | YES | YES | YES | NO |
| B189 | CKD | YES | NO | YES | YES | NO | NO | NO | YES | YES | NO | YES |
| B190 | CKD | YES | NO | NO | NO | NO | NO | NO | YES | YES | YES | NO |
| B192 | CKD | NO | NO | YES | NO | YES | NO | YES | NO | NO | NO | YES |
| B193 | CKD | NO | YES | YES | YES | YES | YES | YES | YES | YES | YES | YES |
| B194 | CKD | YES | NO | YES | YES | YES | YES | YES | YES | YES | YES | YES |
| D2 | W-CKD | YES | NO | YES | NO | YES | NO | NO | YES | YES | NO | NO |
| D9 | W-CKD | YES | YES | YES | NO | YES | YES | YES | YES | YES | NO | NO |
| D10 | W-CKD | YES | NO | YES | YES | NO | NO | YES | YES | YES | NO | YES |
| D16 | W-CKD | YES | NO | YES | YES | YES | NO | NO | YES | YES | NO | NO |
| D21 | W-CKD | YES | NO | YES | YES | YES | YES | YES | YES | YES | YES | YES |
| D24 | W-CKD | NO | NO | NO | NO | NO | NO | NO | YES | YES | NO | NO |
| D25 | W-CKD | YES | NO | YES | NO | YES | NO | NO | YES | YES | NO | YES |
| D65 | W-CKD | YES | YES | YES | YES | YES | YES | YES | YES | YES | NO | NO |
| D68 | W-CKD | NO | NO | NO | NO | NO | YES | YES | YES | NO | NO | NO |
| D70 | W-CKD | NO | NO | NO | NO | YES | YES | YES | YES | YES | NO | NO |
| D73 | W-CKD | YES | YES | YES | YES | YES | YES | YES | YES | NO | YES | NO |
| D74 | W-CKD | YES | YES | YES | YES | YES | YES | YES | YES | YES | YES | NO |
| D84 | W-CKD | YES | YES | YES | YES | YES | YES | YES | YES | YES | NO | YES |
| D85 | W-CKD | YES | NO | YES | YES | NO | YES | NO | YES | YES | NO | NO |
| D89 | W-CKD | YES | NO | YES | YES | NO | YES | YES | YES | NO | NO | NO |
| D90 | W-CKD | YES | NO | YES | YES | NO | NO | YES | YES | YES | NO | NO |
| D91 | W-CKD | YES | NO | YES | YES | NO | YES | NO | YES | YES | NO | NO |
| D95 | W-CKD | YES | NO | NO | NO | YES | YES | YES | YES | YES | YES | NO |
| D96 | W-CKD | NO | NO | YES | NO | YES | YES | YES | YES | YES | YES | NO |
| D101 | W-CKD | YES | NO | YES | NO | YES | YES | YES | YES | YES | NO | NO |
| D108 | W-CKD | YES | NO | NO | NO | NO | NO | NO | YES | NO | NO | NO |
| D110 | W-CKD | YES | YES | YES | NO | YES | YES | YES | YES | YES | NO | NO |
| D111 | W-CKD | YES | YES | YES | YES | YES | YES | NO | NO | NO | YES | NO |
| D120 | W-CKD | NO | NO | NO | NO | NO | NO | NO | YES | NO | NO | NO |
| D121 | W-CKD | YES | NO | YES | NO | YES | YES | YES | YES | YES | NO | NO |
| D125 | W-CKD | NO | NO | YES | NO | YES | YES | YES | YES | NO | YES | NO |
| D128 | W-CKD | YES | NO | YES | NO | YES | YES | YES | YES | YES | NO | NO |
| D133 | W-CKD | YES | NO | NO | YES | NO | NO | YES | YES | YES | NO | NO |
| D147 | W-CKD | YES | YES | NO | YES | NO | YES | NO | YES | YES | NO | NO |
| D150 | W-CKD | YES | NO | YES | NO | YES | YES | YES | YES | YES | YES | NO |
| D151 | W-CKD | YES | NO | YES | NO | YES | YES | YES | YES | YES | YES | NO |
| D155 | W-CKD | YES | NO | NO | NO | NO | YES | NO | NO | YES | NO | NO |
| D159 | W-CKD | YES | NO | NO | YES | YES | YES | YES | YES | YES | YES | NO |
| D160 | W-CKD | YES | NO | YES | NO | YES | YES | YES | YES | YES | YES | NO |
| D173 | W-CKD | YES | NO | NO | YES | YES | YES | YES | YES | YES | YES | NO |

**Legend**: Code with B: Chronic kidney disease patients; code with D: without chronic kidney disease patients; CKD: Chronic kidney disease; W-CKD: without chronic kidney disease; AMC: amoxicillin+ clavulanic acid; AMX: amoxicillin; CRO: ceftriaxone; CTX: cefotaxime; FEP: cefepime; ATM: aztreonam; IMP: imipenem*.*

| S8. The multidrug resistance status of pathotypes of *Escherichia coli* isolates. | | | | | |
| --- | --- | --- | --- | --- | --- |
| **CODES** | **CKD_**  **Status** | **EPEC (*BfpA*)** | **STEC (*Stx*)** | **ETEC (*LT*)** | **Multidrug_**  **resistance_profile** |
| B2 | CKD | YES | NO | NO | MDR |
| B3 | CKD | YES | NO | NO | MDR |
| B7 | CKD | YES | NO | NO | MDR |
| B12 | CKD | YES | NO | NO | MDR |
| B17 | CKD | YES | NO | NO | MDR |
| B18 | CKD | YES | NO | NO | MDR |
| B19 | CKD | YES | NO | NO | MDR |
| B20 | CKD | YES | NO | NO | MDR |
| B22 | CKD | NO | YES | NO | MDR |
| B24 | CKD | NO | NO | YES | MDR |
| B26 | CKD | YES | NO | NO | MDR |
| B27 | CKD | NO | YES | NO | MDR |
| B30 | CKD | NO | YES | NO | MDR |
| B32 | CKD | YES | NO | NO | MDR |
| B34 | CKD | YES | NO | NO | MDR |
| B39 | CKD | NO | YES | NO | MDR |
| B40 | CKD | NO | NO | YES | MDR |
| B41 | CKD | NO | NO | YES | MDR |
| B44 | CKD | NO | YES | NO | MDR |
| B45 | CKD | NO | YES | NO | MDR |
| B46 | CKD | NO | NO | YES | MDR |
| B47 | CKD | NO | YES | NO | MDR |
| B50 | CKD | NO | NO | YES | MDR |
| B51 | CKD | NO | NO | YES | MDR |
| B52 | CKD | YES | NO | NO | MDR |
| B55 | CKD | YES | NO | NO | MDR |
| B66 | CKD | NO | NO | YES | MDR |
| B73 | CKD | NO | YES | NO | MDR |
| B78 | CKD | YES | NO | NO | MDR |
| B84 | CKD | NO | YES | NO | MDR |
| B87 | CKD | NO | YES | NO | MDR |
| B91 | CKD | NO | NO | YES | MDR |
| B92 | CKD | NO | NO | YES | MDR |
| B102 | CKD | NO | YES | NO | MDR |
| B103 | CKD | YES | NO | NO | MDR |
| B115 | CKD | YES | NO | NO | MDR |
| B116 | CKD | NO | NO | YES | MDR |
| B125 | CKD | NO | YES | NO | MDR |
| B126 | CKD | YES | NO | NO | MDR |
| B130 | CKD | NO | YES | NO | MDR |
| B132 | CKD | NO | NO | YES | MDR |
| B133 | CKD | YES | NO | NO | MDR |
| B140 | CKD | NO | NO | YES | MDR |
| B144 | CKD | NO | YES | NO | MDR |
| B155 | CKD | YES | NO | NO | MDR |
| B157 | CKD | YES | NO | NO | MDR |
| B160 | CKD | YES | NO | NO | MDR |
| B161 | CKD | NO | YES | NO | MDR |
| B163 | CKD | NO | NO | YES | MDR |
| B169 | CKD | YES | NO | NO | MDR |
| B171 | CKD | NO | NO | YES | MDR |
| B172 | CKD | YES | NO | NO | MDR |
| B173 | CKD | NO | YES | NO | MDR |
| B174 | CKD | YES | NO | NO | MDR |
| B180 | CKD | NO | NO | YES | MDR |
| B181 | CKD | NO | YES | NO | MDR |
| B183 | CKD | YES | NO | NO | MDR |
| B184 | CKD | YES | NO | NO | MDR |
| B189 | CKD | YES | NO | NO | MDR |
| B190 | CKD | YES | NO | NO | MDR |
| B192 | CKD | NO | YES | NO | MDR |
| B193 | CKD | YES | NO | NO | MDR |
| B194 | CKD | YES | NO | NO | MDR |
| D2 | W-CKD | NO | NO | YES | MDR |
| D9 | W-CKD | YES | NO | NO | MDR |
| D10 | W-CKD | NO | NO | YES | MDR |
| D16 | W-CKD | NO | YES | NO | MDR |
| D21 | W-CKD | YES | NO | NO | MDR |
| D24 | W-CKD | YES | NO | NO | N-MDR |
| D25 | W-CKD | NO | NO | YES | MDR |
| D65 | W-CKD | NO | NO | YES | MDR |
| D68 | W-CKD | NO | YES | NO | N-MDR |
| D70 | W-CKD | NO | YES | NO | N-MDR |
| D73 | W-CKD | NO | NO | YES | MDR |
| D74 | W-CKD | YES | NO | NO | MDR |
| D84 | W-CKD | NO | NO | YES | MDR |
| D85 | W-CKD | NO | YES | NO | MDR |
| D89 | W-CKD | NO | YES | NO | MDR |
| D90 | W-CKD | NO | YES | NO | MDR |
| D91 | W-CKD | NO | NO | YES | MDR |
| D95 | W-CKD | YES | NO | NO | MDR |
| D96 | W-CKD | YES | NO | NO | MDR |
| D101 | W-CKD | NO | NO | YES | MDR |
| D108 | W-CKD | NO | YES | NO | N-MDR |
| D110 | W-CKD | NO | NO | YES | MDR |
| D111 | W-CKD | NO | YES | NO | MDR |
| D120 | W-CKD | NO | YES | NO | N-MDR |
| D121 | W-CKD | NO | NO | YES | MDR |
| D125 | W-CKD | NO | NO | YES | MDR |
| D128 | W-CKD | NO | NO | YES | MDR |
| D133 | W-CKD | NO | YES | NO | MDR |
| D147 | W-CKD | NO | NO | YES | MDR |
| D150 | W-CKD | YES | NO | NO | MDR |
| D151 | W-CKD | YES | NO | NO | MDR |
| D155 | W-CKD | YES | NO | NO | MDR |
| D159 | W-CKD | NO | YES | NO | MDR |
| D160 | W-CKD | YES | NO | NO | MDR |
| D173 | W-CKD | NO | NO | YES | MDR |

**Legend**: Code with B: Chronic kidney disease patients; code with D: without chronic kidney disease patients; CKD: Chronic kidney disease; W-CKD: without chronic kidney disease; EPEC: Enteropathogenic*E. coli;* ETEC: Enterotoxigenic*E. coli;* STEC: Shiga toxin producing *E. coli,* MDR: multidrug resistance; N-MDR: non multidrug-resistance.
